# FGFR2 Promotes Breast Tumorigenicity through Maintenance of Breast Tumor-Initiating Cells

**DOI:** 10.1371/journal.pone.0051671

**Published:** 2013-01-02

**Authors:** Sungeun Kim, Anna Dubrovska, Richard J. Salamone, John R. Walker, Kathryn B. Grandinetti, Ghislain M. C. Bonamy, Anthony P. Orth, Jimmy Elliott, Diana Graus Porta, Carlos Garcia-Echeverria, Venkateshwar A. Reddy

**Affiliations:** 1 Genomics Institute of the Novartis Research Foundation, San Diego, California, United States of America; 2 The Scripps Research Institute, La Jolla, California, United States of America; 3 Novartis Institutes for BioMedical Research, Novartis Pharma AG, Basel, Switzerland; Wayne State University School of Medicine, United States of America

## Abstract

Emerging evidence suggests that some cancers contain a population of stem-like TICs (tumor-initiating cells) and eliminating TICs may offer a new strategy to develop successful anti-cancer therapies. As molecular mechanisms underlying the maintenance of the TIC pool are poorly understood, the development of TIC-specific therapeutics remains a major challenge. We first identified and characterized TICs and non-TICs isolated from a mouse breast cancer model. TICs displayed increased tumorigenic potential, self-renewal, heterogeneous differentiation, and bipotency. Gene expression analysis and immunostaining of TICs and non-TICs revealed that FGFR2 was preferentially expressed in TICs. Loss of FGFR2 impaired self-renewal of TICs, thus resulting in marked decreases in the TIC population and tumorigenic potential. Restoration of FGFR2 rescued the defects in TIC pool maintenance, bipotency, and breast tumor growth driven by FGFR2 knockdown. In addition, pharmacological inhibition of FGFR2 kinase activity led to a decrease in the TIC population which resulted in suppression of breast tumor growth. Moreover, human breast TICs isolated from patient tumor samples were found enriched in a FGFR2+ population that was sufficient to initiate tumor growth. Our data suggest that FGFR2 is essential in sustaining the breast TIC pool through promotion of self-renewal and maintenance of bipotent TICs, and raise the possibility of FGFR2 inhibition as a strategy for anti-cancer therapy by eradicating breast TICs.

## Introduction

Stem cells may play an essential role not only in regenerative capacity, but also in the development of cancer [1]. The unique property of stem cells to self-renew must be tightly regulated to obey genetic restriction and meet their environmental needs. Dysregulation of self-renewal may be a key event underlying cancer. When breast undergoes changes of tissue renewal and massive tissue expansion during pregnancy, stem cells in the invading tips of mammary glands, known as terminal end buds (TEBs) are believed to be responsible for these cellular dynamics [2]. The abilities of stem cells in TEBs to extensively proliferate and invade different regions of the organ resemble those of solid tumors.

The notion that tumors are heterogeneous and that tumor cells share certain properties with normal stem cells led to the hypothesis that tumors may contain a subset of self-renewing, stem-like tumorigenic cells, TICs, which drive tumor initiation and growth. In this hypothesis, only the tumor initiating cells are capable of unlimited self-renewal, extensive proliferation, and give rise to heterogeneous progenies while differentiated progenies have a limited proliferative potential [3], [4]. The demonstration of TICs in many types of cancer including leukemia, breast, brain, colon, skin, head and neck, liver, and pancreatic cancer supports the concept of tumor hierarchy [5]–[13]. Furthermore, TICs have been proposed to be responsible for tumor recurrence. Based on this view, therapeutic strategies for selectively eradicating tumor-initiating cells should lead to successful curative therapies for cancer. However, there is little evidence to support this concept, mainly due to the poor understanding of the molecular mechanisms underlying tumor initiation and the stem-like function of TICs.

Mouse models have been attractive tools to study tumor-initiating cells with their unlimited transplantation assays in many types of cancer [14]–[16]. The recent evidence suggests that the different environments between mice and human can influence the xenotransplantation assay in which human tumor cells that are able to survive in a foreign environment may be selected [17], [18]. Therefore, using mice models we can avoid some of the intrinsic problems of measuring stemness through human into mouse xenotransplantation.

To understand the molecular mechanisms underlying tumor initiation and stem-like properties of TICs, we investigated to identify the regulators that are critical for maintenance of a TIC pool. To do so, we used a mouse mammary tumor virus-polyoma middle T (MMTV-PyMT) transgenic mouse, which is a model of human breast cancer with distinct stages of tumor progression from premalignant stage to malignant carcinomas [19], [20]. The cellular origins of this breast tumor that can self-renew and drive tumor initiation are uncertain. The ability to isolate and characterize TICs allowed us to compare gene expression and biological functions between stem-like TICs and differentiated non-TICs. This comparison and functional studies with genetic manipulations demonstrated that FGFR2 is essential in sustaining the breast TIC pool through promotion of self-renewal and inhibition of differentiation of TICs. Moreover, we found human breast TICs enriched in a FGFR2+ population which was sufficient to initiate tumor growth.

## Results

### Breast Tumor Initiating Cells Are Highly Enriched in the CD29^high^CD24^+^ Population

**Figure 1:**
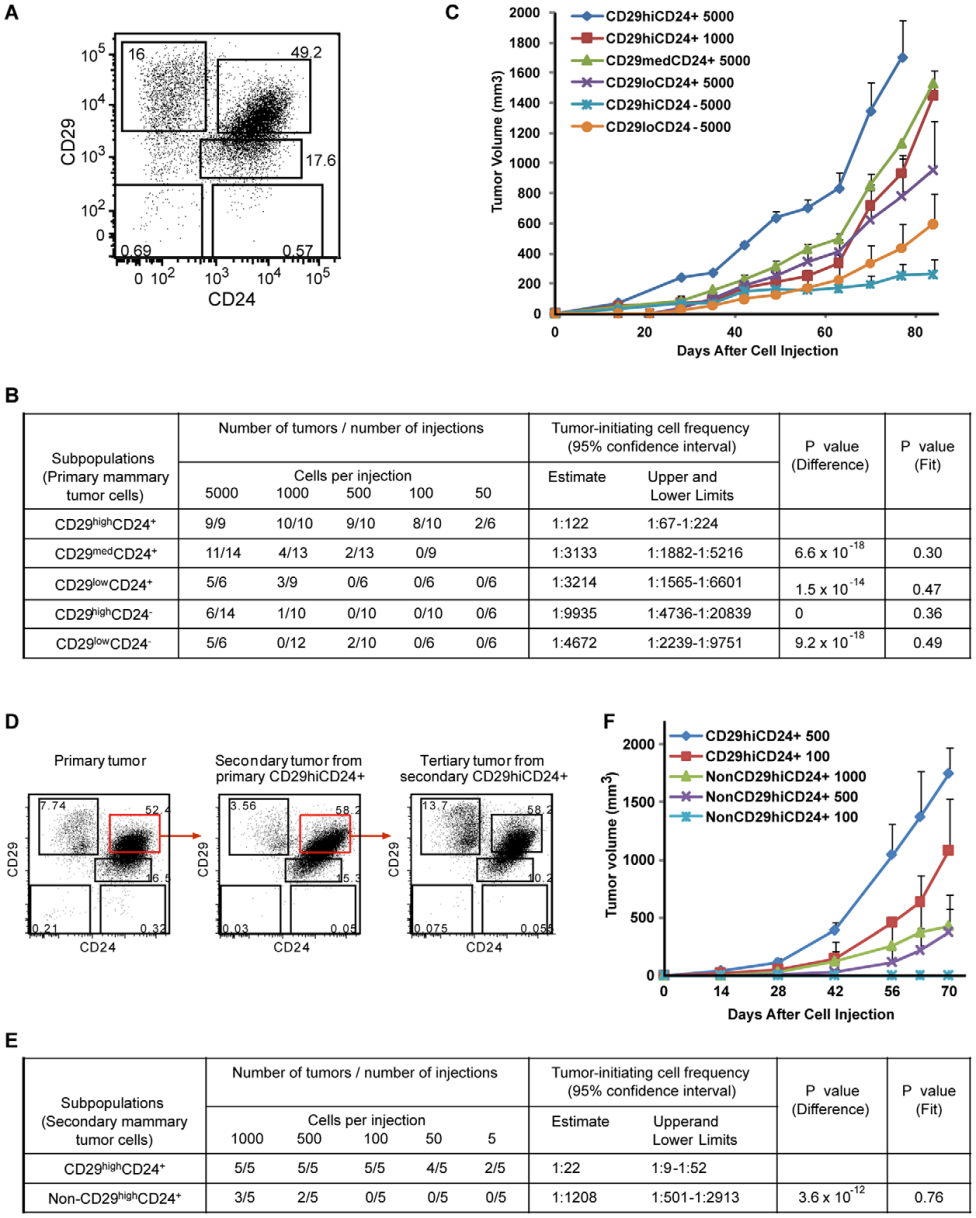
Enrichment of Breast Tumor-Initiating Cells in the CD29^hi^CD24^+^ Population. (A) Isolation of five populations from MMTV-PyMT breast tumors based on the expression of cell-surface markers CD29 and CD24 by FACS. (B) Limiting dilution analyses of tumors to calculate the frequency of TICs in primary breast tumor subpopulations isolated from the primary tumors during secondary tumor formation. The number of tumors was measured 12–16 weeks after injection of 50–5000 tumor cells of five subpopulations into the breast of NOD/SCID mice. The significance of difference in TIC frequencies for each subpopulations against the CD29^high^CD24^+^ population is indicated by *P* values (*P difference*<0.05). The single hit hypothesis was validated by likelihood ratio tests and accepted for all dilution series (*P fit*>0.05). (C) Comparison of the growth of secondary breast tumors which arose from purified primary tumor cells of different subpopulations. The number of cells injected is indicated next to the population. (D) Flow cytometry analysis of CD24 and CD29 expression after serial transplantation demonstrates that secondary and tertiary tumors initiated from the primary and secondary CD29^hi^CD24^+^ population, respectively are as heterogeneous as the primary tumors. (E) Limiting dilution analyses of tumors to calculate the frequency of TICs in secondary subpopulations isolated from the secondary tumors during tertiary tumor formation. The CD29^high^CD24^+^ population isolated from the secondary tumors displayed significantly (*P = *3.6×10^−12^) higher frequency of TICs compared to non-CD29^high^CD24^+^ populations. (F) Comparison of the growth of tertiary breast tumors in NOD/SCID mice which were generated from purified tumor cells of the secondary CD29^high^CD24^+^ or non-CD29^high^CD24^+^ populations.

To understand the molecular mechanisms underlying tumor initiation and self-renewal, we first identified potential TIC and non-TIC populations that were isolated from breast tumors of MMTV-PyMT transgenic mice. Given that many functional properties of normal stem cells are shared by TICs, we isolated and examined subpopulations from the breast tumors, based on expression of cell-surface markers CD29 and CD24, which were used to enrich for mouse normal mammary stem cells [21]. Hematopoietic and endothelial cell lineages from the tumor cells were depleted by staining with antibodies against CD45, TER119 and CD31. Five subpopulations (CD29^high^CD24^+^, CD29^med^CD24^+^, CD29^low^CD24^+^, CD29^high^CD24^−^, and CD29^low^CD24^−^) were gated and isolated from the breast tumors by fluorescence-activated cell sorting (FACS), and the purity of the sorted populations was assessed by FACS analysis (Figure 1A and S1). To determine the frequency of TICs in these subpopulations, we performed limiting dilution analyses of tumors by injecting the purified tumor cells in decreasing numbers into NOD/SCID mice (Figure 1B). The calculated frequency of TICs in the CD29^high^CD24^+^ subpopulation was ∼1 in 122, whereas the frequencies of TICs in the other four subpopulations (CD29^med^CD24^+^, CD29^low^CD24^+^, CD29^high^CD24^−^, and CD29^low^CD24^−^) were as low as 1 in 3,133 to 1 in 9,935 (Figure 1B), indicating that breast tumor initiating cells are highly enriched in the CD29^high^CD24^+^ subpopulation.

Limited tumor growth from the CD29^med^CD24^+^, CD29^low^CD24^+^, CD29^high^CD24^−^, and CD29^low^CD24^−^ subpopulations was observed when a higher number of cells was injected (Figure 1C). Tumors derived from CD29^high^CD24^+^ population grew faster than other four subpopulations even with low number of cells implanted. For example, tumor growth from 1,000 to 5,000 cells of CD29^high^CD24^+^ population was faster than that from 5,000 cells of most of the other subpopulations (Figure 1C). In particular, tumor growth from even 500 CD29^high^CD24^+^ cells was faster than that from 1,000 to 5,000 cells from the other four subpopulations (Figure S2). These results suggest that the CD29^high^CD24^+^ subpopulation is enriched for TICs and has significantly increased tumorigenic potential compared with the other four subpopulations.

### CD29^high^CD24^+^ Cells Have Self-Renewal and Heterogeneous Differentiation Potentials

To determine whether tumor cells in the CD29^high^CD24^+^ population can self-renew and generate heterogeneous progenies, tumors generated from the CD29^high^CD24^+^ population were examined by flow cytometry and serial transplantation. The secondary tumors arising from the primary CD29^high^CD24^+^ population were FACS-sorted into CD29^high^CD24^+^ and non-CD29^high^CD24^+^ (all other four) populations, and then tertiary tumors were generated from the transplanted secondary CD29^high^CD24^+^ population (Figure 1D). Secondary and tertiary tumors arising from the primary and secondary CD29^high^CD24^+^ population, respectively, contained heterogeneous populations that were originally seen in the primary tumors (Figure 1D). Limiting dilution analyses of tumors derived from as few as 5 cells revealed that the calculated frequency of TICs in the secondary CD29^high^CD24^+^ population was increased to ∼1 in 22, whereas the frequency of TICs in the secondary non-CD29^high^CD24^+^ population was as low as ∼1 in 1,208 (Figure 1E). Importantly, the significant increase in TIC frequency (∼1 in 22) in the secondary CD29^high^CD24^+^ population compared to that (∼1 in 122) in the primary CD29^high^CD24^+^ population (Figure 1B) suggests that more tumor initiating cells were generated in the CD29^high^CD24^+^ population through serial transplantations, indicating self-renewing capability of the TICs in the CD29^high^CD24^+^ cells. Very limited tertiary tumor growth from secondary non-CD29^high^CD24^+^ population was evident compared with the CD29^high^CD24^+^ population (Figure 1F). The tertiary tumor growth from 1,000 secondary non-CD29^high^CD24^−^ cells was slower than that from 100 secondary CD29^high^CD24^+^ cells (Figure 1F). These results suggest that the TICs in the CD29^high^CD24^+^ population are capable of self-renewal and heterogeneous differentiation, indicative of stem cell-like properties.

**Figure 2:**
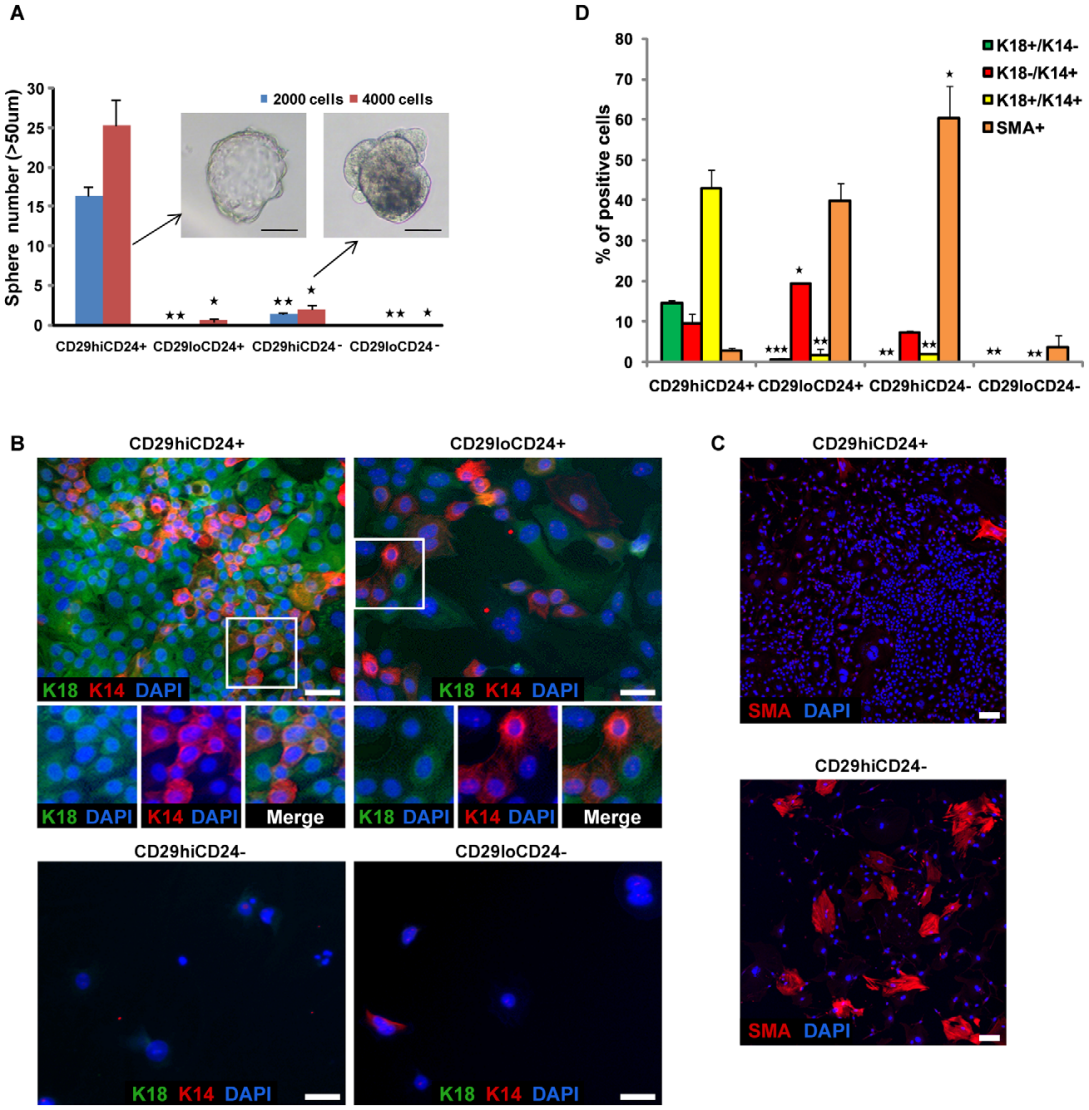
CD29^high^CD24^+^ Cells Have Self-Renewal Capacity and Contain Bipotent Precursor-like Cells. (A) Mammosphere assay for the four FACS-sorted subpopulations from MMTV-PyMT breast tumors. The number of spheres (>50 µm in diameter) formed from 2000 or 4000 seeded cells was measured 3 weeks after seeding. Statistical comparison with CD29^high^CD24^+^ subpopulation (*P<0.02; **P<0.01). Data (n = 3) represent mean ± SEM. Scale bars in the sphere images represent 50 µm. (B) Differentiation potential of breast tumor subpopulations assessed by immunofluorescence. The breast tumor cells from the four FACS-sorted subpopulations were cultured under the differentiation condition. The sorted cells from various populations were plated at the same cell density on collagen-coated plates. The differentiated cells were stained for the luminal epithelial marker (K18, green), the myoepithelial markers (K14 and SMA, red), and DAPI (nuclei, blue). A significant portion of CD29^high^CD24^+^ cells contains K18^+^K14^+^ (bipotent precursor-like), whereas the majority of cells from the other subpopulations contain lineage-restricted cells. Magnifications of the boxed regions are shown below the figure. Scale bars represent 32 µm. (C) Myoepithelial lineage-specific differentiation assessed by immunofluorescence. The differentiated tumor cells from FACS-sorted subpopulations were stained for myoepithelial markers (SMA, red), and DAPI (nuclei, blue). Scale bars represent 120 µm. (D) Quantification of the immunofluorescence images (B and C) for the frequency of K18^+^K14^−^ (luminal), K18^−^K14^+^ (myoepithelial), K18^+^K14^+^ (bipotent precursor-like), and SMA^+^ (myoepithelial) cells. Image quantification was achieved using the multi-wavelength cell scoring analysis module on the ImageXpress Ultra confocal microscope. An average of three values from three images (different positions) per group is presented. Statistical comparison with CD29^high^CD24^+^ subpopulation (*P≤0.05; **P≤0.01; ***P<0.001). Data (n = 3) represent mean ± SEM.

To reconfirm the self-renewal capability of the CD29^high^CD24^+^ tumor cells, we cultured the primary tumor cells isolated from different subpopulations and subjected them to a mammosphere assay in which self-renewing cells can proliferate as three-dimensional floating cell clusters (mammospheres) under nonadherent conditions [22]. The primary tumor cells were cultured at a clonal density to ensure that each mammosphere was generated from a single cell. The CD29^high^CD24^+^ cells displayed markedly (*P*<0.02) higher ability to form mammospheres (>50 µm in diameter) than tumor cells from the other subpopulations (Figure 2A). The few spheres that formed from the CD29^high^CD24^−^ cells were mostly necrotic (Figure 2A). Approximately 16 mammospheres were formed per 2000 seeded cells in the CD29^high^CD24^+^ subpopulation, indicating that 1 in 125 cells can form mammospheres. This frequency (1∶125) of mammosphere-forming cells in the CD29^high^CD24^+^ population was similar to the frequency (1∶122) of TICs in that primary population, calculated by limiting dilution analyses of tumors (Figure 1B). These results suggest that the CD29^high^CD24^+^ population is enriched for cells that show increased self-renewal activity compared to the other populations.

Bipotent precursor cells present in mammary stem/progenitor cells and breast TICs/cancer stem cells have been characterized by coexpression of luminal epithelial cytokeratin 18 (K18)/K8 markers and myoepithelial cytokeratin 14 (K14)/α-smooth muscle actin (SMA) markers [21]–[29]. To investigate whether the CD29^high^CD24^+^ population contains these bipotent precursor-like cells, the breast tumor cells from the four subpopulations were cultured under differentiation conditions and stained for K18, K14, and SMA (Figure 2B, 2C, and S5). Quantification of immunofluorescence images revealed that the frequency of bipotent precursor-like cells expressing both K18 and K14 (K18^+^K14^+^) was significantly higher in the CD29^high^CD24^+^ population (∼43%) compared with the other populations (0–2%) (Figure 2B, 2D, and S5; *P*<0.01). The CD29^high^CD24^+^ population also gave rise to either the luminal (K18^+^K14^−^) or the myoepithelial (K18^−^K14^+^) cells reflecting its bipotent capacity (Figure 2B, 2D, and S5). In contrast, the majority of cells derived from the other three subpopulations including CD29^high^CD24^−^, CD29^low^CD24^+^, and CD29^low^CD24^−^ cells expressed the myoepithelial markers (K18^−^K14^+^ and SMA^+^), indicating that most of them are terminally differentiated into the myoepithelial lineage (Figure 2B, 2C, 2D, and S5). These results, together with those seen in the serial transplantation assays, demonstrate that cells within the CD29^high^CD24^+^ population can drive tumor initiation, maintain the TIC pool through self-renewal, generate heterogeneous progenies, and contain bipotent precursor-like cells, whereas the other subpopulations fail to do so. These data provide further evidence that the CD29^high^CD24^+^ cells possess key attributes of tumor-initiating cells (TICs). Hereafter, we apply the term ‘TICs’ to the CD29^high^CD24^+^ cells.

### FGFR2 Is Preferentially Expressed in Breast TICs

**Figure 3:**
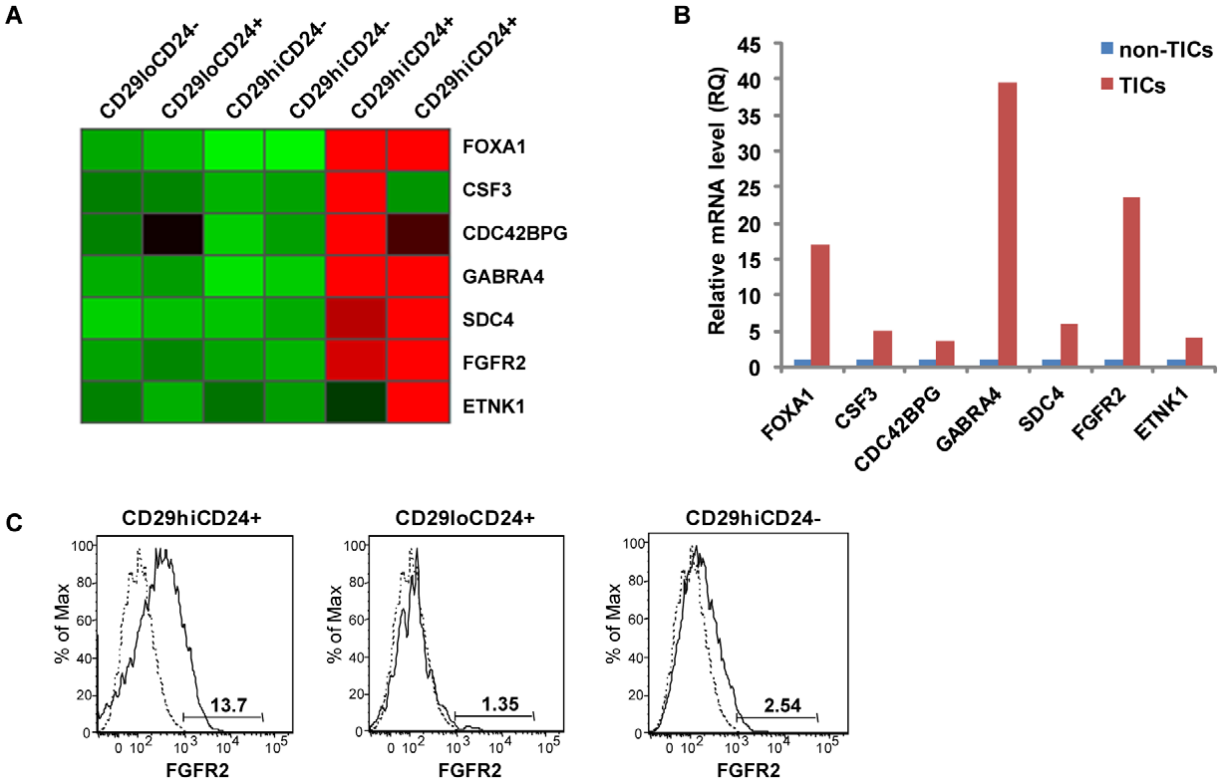
Gene Expression Profiles of Breast TICs and Non-TICs Revealed that FGFR2 Is Preferentially Expressed in Breast TICs. (A) Heat map of microarray analysis depicting the expression levels of the mRNAs of 7 genes that are significantly upregulated in the TIC population compared with the three other non-TIC populations. RNA was prepared from the four FACS-sorted populations of primary MMTV-PyMT breast tumors. Red and green indicate high and low mRNA expression levels, respectively. The expression values were normalized by Z score. (B) The expression levels of the mRNAs of 7 genes in TICs and non-TICs, as determined by quantitative real-time PCR. cDNA isolated from non-TICs was used to normalize data for each primer of gene and generate RQ (relative quantity). (C) The expression levels FGFR2 protein in the subpopulations by flow cytometry. This figure represents a typical result of three independent experiments. Dashed line shows isotype labeling.

To identify genes that regulate stem-like properties in TICs, we analyzed gene expression profiles of stem-like TICs in comparison with lineage-restricted non-TICs. Microarray analysis was performed to examine the expression levels of mRNAs in a TIC (CD29^high^CD24^+^) and three non-TIC populations (CD29^low^CD24^+^, CD29^high^CD24^−^ and CD29^low^CD24^−^) that were FACS-sorted from primary MMTV-PyMT tumors. The fold changes of expression values in the TIC population compared to the three non-TIC populations were analyzed (for microarray data filtering, see materials and methods). This analysis led to the identification of seven genes of which the mRNA levels are markedly upregulated in TICs compared to non-TICs: syndecan 4 (*SDC4*), gamma-aminobutyric acid A receptor subunit alpha 4 (*GABRA4*), fibroblast growth factor receptor 2 (*FGFR2*), colony stimulating factor 3 (*CSF3*), Forkhead-box A1 (*FOXA1*), ethanolamine kinase 1 (*ETNK1*), and CDC42 binding protein kinase gamma (*CDC42BPG*) (Figure 3A). While these genes have not been directly examined for their relationship to stem-like properties of tumor-initiating cells, the majority of these genes have been suggested to be implicated in development, stem cell activities and mammary carcinogenesis [30]–[36]. We further validated the mRNA levels of the seven genes in TICs and non-TICs using quantitative real-time PCR (Figure 3B). TICs exhibited significantly higher (17 to 39-fold) expression of mRNAs of *FOXA1*, *FGFR2*, and *GABRA4* compared to non-TICs.

Recent studies have shown that FGFR2, which encodes a receptor tyrosine kinase, is critical for the maintenance and proliferation of terminal end buds (TEBs), where stem cells are found to be active during mammary gland development [37], [38]. FGFR2 also may play an important role in human breast cancer [32], [39]–[41]. However, the mechanism by which *FGFR2* functions as a risk factor in breast cancer remains unknown. Given our expression profiles displaying the high expression of FGFR2 in breast TICs, we investigated the role of FGFR2 in tumor initiation through the self-renewal and maintenance of breast TICs.

To determine whether the upregulated expression of *FGFR2* mRNA in TICs (microarray data and Figure 3B) corresponds to an increased level of FGFR2 protein, we analyzed the expression levels of FGFR2 protein in TICs and non-TICs. Flow cytometry analysis revealed that expression of FGFR2 protein is higher (5.4 to 10.2-fold) in TICs compared to CD29^low^CD24^+^ and CD29^high^CD24^−^ cells (Figure 3C).

### Loss of FGFR2 Suppresses Breast Tumor Growth and Inhibits Oncogenic Signaling

**Figure 4:**
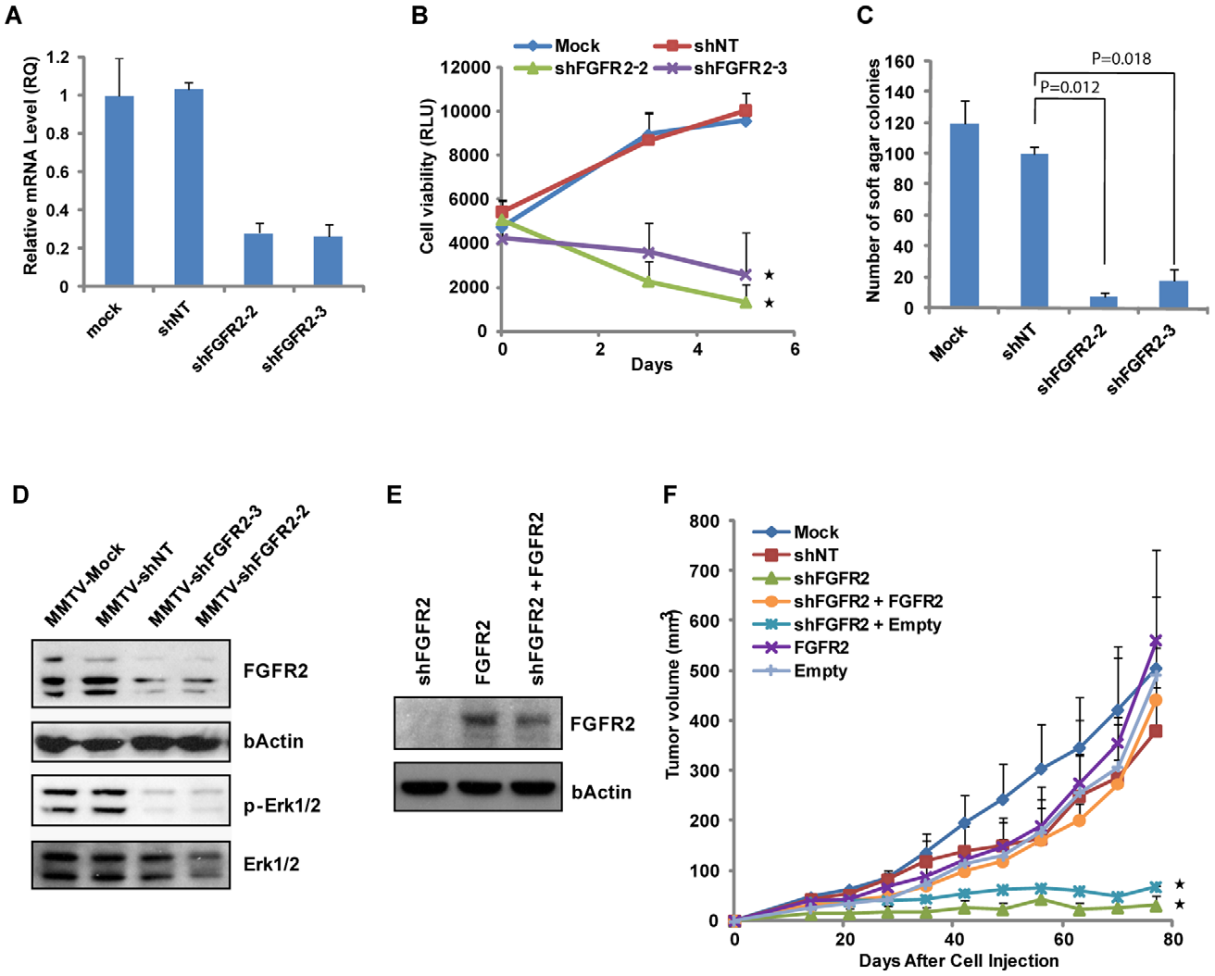
Suppression of Breast Tumor Growth and Inhibition of Oncogenic Signaling by Loss of FGFR2. (A) Quantitative real-time PCR analysis of expression levels of FGFR2 mRNA in MMTV-PyMT breast tumor cells stably transduced with lentiviral shRNAs targeting FGFR2 (shFGFR2) and non-targeting shRNA (shNT). shFGFR2-2 and shFGFR2-3, targeting different regions of *FGFR2* gene, were used for knockdown of FGFR2. Mock (no infection) and shNT infection were used as negative controls. (B) Reduced proliferation of breast tumor cells upon FGFR2 knockdown *in vitro*. Proliferation of tumor cells was determined by cell viability using CellTiter-Glo reagent (Promega). Statistical comparison with shNT (*P<0.05). (C) Suppression of anchorage-independent growth (mean ± SEM., n = 2) of breast tumor cells upon FGFR2 loss was assessed by the ability of cells forming colonies (>100 µm in diameter) in soft agar. The number of colonies from 10000 cells seeded is shown. (D) Inhibition of downstream target activation upon FGFR2 knockdown. shFGFR2 efficiently knocked down the expression of FGFR2 protein and strongly inhibited phosphorylation of Erk1/2, as evidenced by immunoblotting with anti-phospho-ERK1/2 (p-ERK1/2). The bands with lower molecular weights are either cleaved forms or alternative spliced variants of endogenous FGFR2 protein (e.g. sliced variants of carboxyl terminal domains). The membranes were reprobed for actin and Erk1/2 as loading controls. (E) Immunoblotting analysis of expression levels of FGFR2 protein in the breast tumor cells transduced with a lentiviral shFGFR2 and/or a retroviral vector encoding FGFR2. Restored FGFR2 expression was shown in the tumor cells transduced with a lentiviral shFGFR2 and a retroviral FGFR2. Anti-FGFR2 immunoblotting was reprobed for actin as a loading control. (F) Effect of FGFR2 loss and restoration on breast tumor growth *in vivo*. The breast tumor cells transduced with the lentiviral shFGFR2 and/or retroviral FGFR2 vector were injected in the mammary fat pad of the NOD/SCID mice. Mock, shNT, and empty vector were used as negative controls for infection, lentiviral shRNA, and retroviral cDNA, respectively. Data (n = 4) represent mean ± SEM. Statistical comparison with shNT (*P<0.037).

To validate FGFR2 oncogenic function in MMTV-PyMT breast tumors, we transduced primary MMTV-PyMT breast tumor cells with lentiviral short hairpin RNAs (shRNAs) targeting FGFR2 (shFGFR2). Two different shRNAs targeting different regions of *FGFR2* displayed greater than 70% knockdown of the FGFR2 mRNA level (Figure 4A) and a strong reduction in the level of FGFR2 protein (Figure 4D) in tumor cells stably transduced with the lentiviral shFGFR2s. The shFGFR2s significantly inhibited cell proliferation *in vitro* (Figure 4B) and anchorage independent growth in soft agar (Figure 4C) as compared to non-targeting shRNA (shNT). This suppressed growth was accompanied by markedly reduced phosphorylation of Erk1/2 MAPK and decreased phosphorylation of FRS2, downstream targets of FGFR2 signaling (Figure 4D and S6), indicating the dependence of downstream oncogenic signaling on FGFR2 expression.

To evaluate the effect of shFGFR2 on breast tumor growth *in vivo*, breast tumor cells transduced with the lentiviral shFGFR2 were injected in the mammary fat pad of NOD/SCID mice. Loss of FGFR2 resulted in marked suppression of breast tumor growth (Figure 4F). To exclude the possibility that the observed growth inhibition was due to off-target effects of the shRNAs, we performed rescue experiments by ectopically expressing shRNA-resistant FGFR2 (Figure 4E) in tumor cells suppressed by shFGFR2. Restoration of FGFR2 rescued the shFGFR2-driven growth defect and was sufficient to re-initiate tumor growth *in vivo* , whereas the empty vector control failed to do so (Figure 4F), suggesting that the antiproliferative effects of shFGFR2 are not off-target effects.

### Loss of FGFR2 Impaired Self-Renewal of Breast TICs and led to a reduced TIC Pool

**Figure 5:**
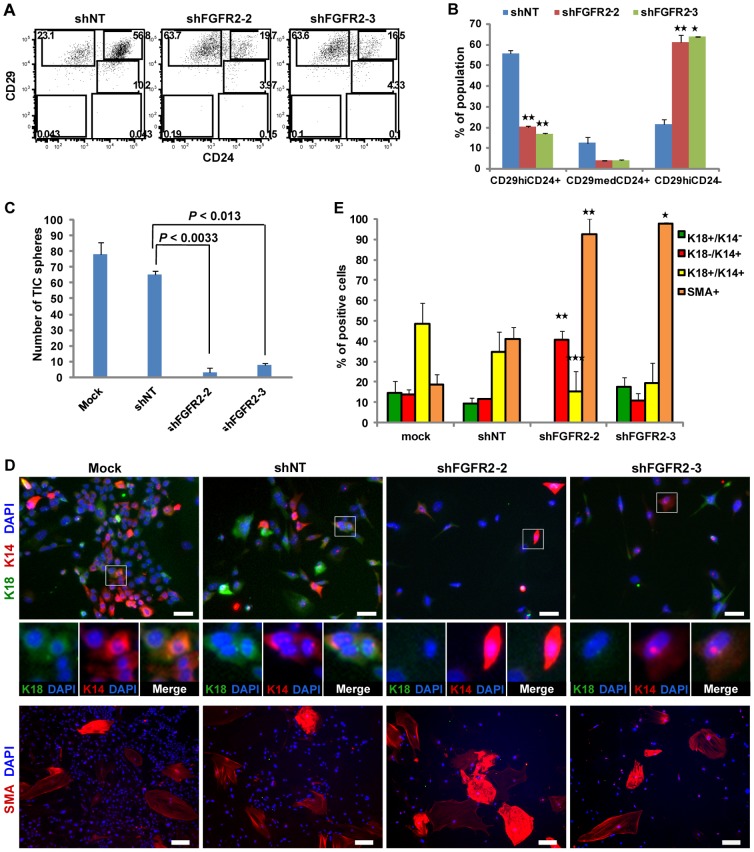
Loss of FGFR2 Impaired Self-Renewal of Breast TICs, Resulting in a Decreased TIC Pool. (A) Flow cytometry analysis of CD24 and CD29 expression for TIC and non-TIC subpopulation frequencies in shRNA-transduced primary MMTV-PyMT breast tumor cells. (B) Quantification of TIC and non-TIC subpopulation frequencies in shRNA transduced breast tumor cells determined by FACS analysis (A). Data (n = 2) represent mean ± s.d. Statistical comparison with shNT (*P<0.027; **P<0.015). (C) Reduced TIC sphere formation (mean ± SEM., n = 2) of breast tumor cells upon FGFR2 knockdown. Ten thousand FACS-sorted TICs were transduced with lentiviral shRNAs and then subjected to a mammosphere (>50 µm in diameter) forming assay. (D) Differentiation potential of shRNA-transduced breast tumor cells as assessed by immunofluorescence. The shRNA-transduced tumor cells were cultured with a selective drug for 7 days after infection and then were plated them at the same cell density under the differentiation condition for 6 days. The cells were stained for the luminal epithelial marker (K18, green), myoepithelial markers (K14 and SMA, red), and DAPI (nuclei, blue). The scale bars for K18/K14 and SMA represent 40 µm and 140 µm, respectively. (E) Quantification of the immunofluorescence images (D) for the frequency of K18^+^K14^−^ (luminal), K18^−^K14^+^ (myoepithelial), K18^+^K14^+^ (bipotent precursor-like), and SMA^+^ (myoepithelial) cells in shRNA transduced tumor cells. Image quantification was achieved using the multi-wavelength cell scoring analysis module on the ImageXpress Ultra confocal microscope. Statistical comparison with mock infection (*P<0.05; **P≤0.01; ***P<0.0003). Data (n = 3) represent mean ± SEM.

To investigate whether the impaired growth of breast tumor cells transduced with shFGFR2 was due to reduced numbers of TICs, we analyzed frequencies of TIC and non-TIC subpopulations in shRNA-transduced breast tumors using flow cytometry. Indeed, shFGFR2-transduced tumor cells exhibited a strong reduction (64 to 70%) in the TIC subpopulation (CD29^high^CD24^+^) compared to shNT-transduced tumor cells (Figure 5A and 5B). In contrast, the shFGFR2-transduced tumor cells displayed a significant increase (65 to 67%) in the non-TIC (CD29^high^CD24^−^) subpopulation compared to shNT-transduced tumor cells (Figure 5A and 5B). These results suggest that loss of FGFR2 leads to decreased numbers of TICs and increased number of lineage-restricted non-TICs.

To determine whether decreased number of TICs upon FGFR2 loss was due to an impaired self-renewal capacity of TICs, we transduced the purified TICs with lentiviral shFGFR2 and then examined their ability to form TIC spheres. The sphere-forming capability of TICs was significantly reduced (8 to 19-fold) by loss of FGFR2 as compared with that of shNT-transduced TICs (Figure 5C).

We next investigated whether loss of FGFR2 affects the differentiation potential of the breast tumor cells. The shRNA-transduced tumor cells were differentiated on collagen-coated plates and then costained for lineage-specific markers K18, K14, and SMA (Figure 5D). The quantification of immunofluorescence images revealed that shFGFR2-transduced tumor cells had ∼3-fold lower frequency of bipotent precursor-like cells (K18^+^K14^+^) compared to the control cells (Figure 5D and 5E). In contrast, the majority of FGFR2-knockdown tumor cells gave rise to the terminally differentiated, myoepithelial lineage (SMA^+^ and K18^−^K14^+^) (Figure 5D and 5E). As the non-TIC populations were shown to enrich for SMA+ cells (Figure 2C and 2D), this data suggest that the increase in SMA+ cells in the FGFR2 shRNA-transfected cells results from the decrease in the TIC population and the increase in the non-TIC population upon FGFR2 knockdown, which is consistent with the flow cytometry data (Figure 5A and 5B).

Furthermore, we examined the differentiation potential of the transplanted tumors *in vivo* upon FGFR2 knockdown. The immunofluorescence staining for K18, K14 and SMA in those tumors revealed that shFGFR2-transduced cells containing enriched non-TIC populations generated the breast tumors with markedly reduced numbers of bipotent precursor-like cells (K18^+^K14^+^) and increased numbers of myoepithelial cells (K18^−^K14^+^ and SMA^+^) (Figure S3A and S3B), compared to the shNT-transduced tumors, indicating terminal differentiation of a majority of tumor cells into myoepithelial lineages in tumors *in vivo*, in accordance with the observation *in vitro* (Figure 5D and 5E). The shNT-transduced tumors contained the bipotent precursor-like cells (K18^+^K14^+^), luminal epithelial cells (K18^+^K14^−^) and myoepithelial cells (K18^−^K14^+^ and SMA^+^) (Figure S3A and S3B). These immunohistology patterns of shNT-transduced tumors were very similar to those of the tumors derived from primary MMTV-PyMT tumor cells. Ectopic expression of FGFR2 in shFGFR2-transduced tumors led to a considerable increase in bipotent precursor-like cells (K18^+^K14^+^) (Figure S3A), suggesting that restoration of FGFR2 rescued the defect in bipotent capacity driven by knockdown of FGFR2. We also examined the frequencies of TIC and non-TIC subpopulations in shRNA-transduced breast tumors by flow cytometry. Tumors transduced with lentiviral shFGFR2 displayed a strong reduction (∼63%) in the TIC subpopulation (CD29^high^CD24^+^) and a significant increase (∼71%) in the non-TIC (CD29^med^CD24^+^) subpopulation compared to shNT-transduced tumors (Figure S3C). Conversely, restoration of FGFR2 in shFGFR2-transduced tumors led to a marked increase (∼60%) in the TIC subpopulation compared to shFGFR2-transduced tumors (Figure S3C). Taken together, these results suggest that FGFR2 plays an important role in sustaining the breast TIC pool through promotion of self-renewal and maintenance of bipotency of TICs.

### Pharmacological Inhibition of FGFR2 Kinase Activity Led to a Decrease in the Breast TIC Population which Resulted in Suppression of Breast Tumor Growth In Vivo

**Figure 6:**
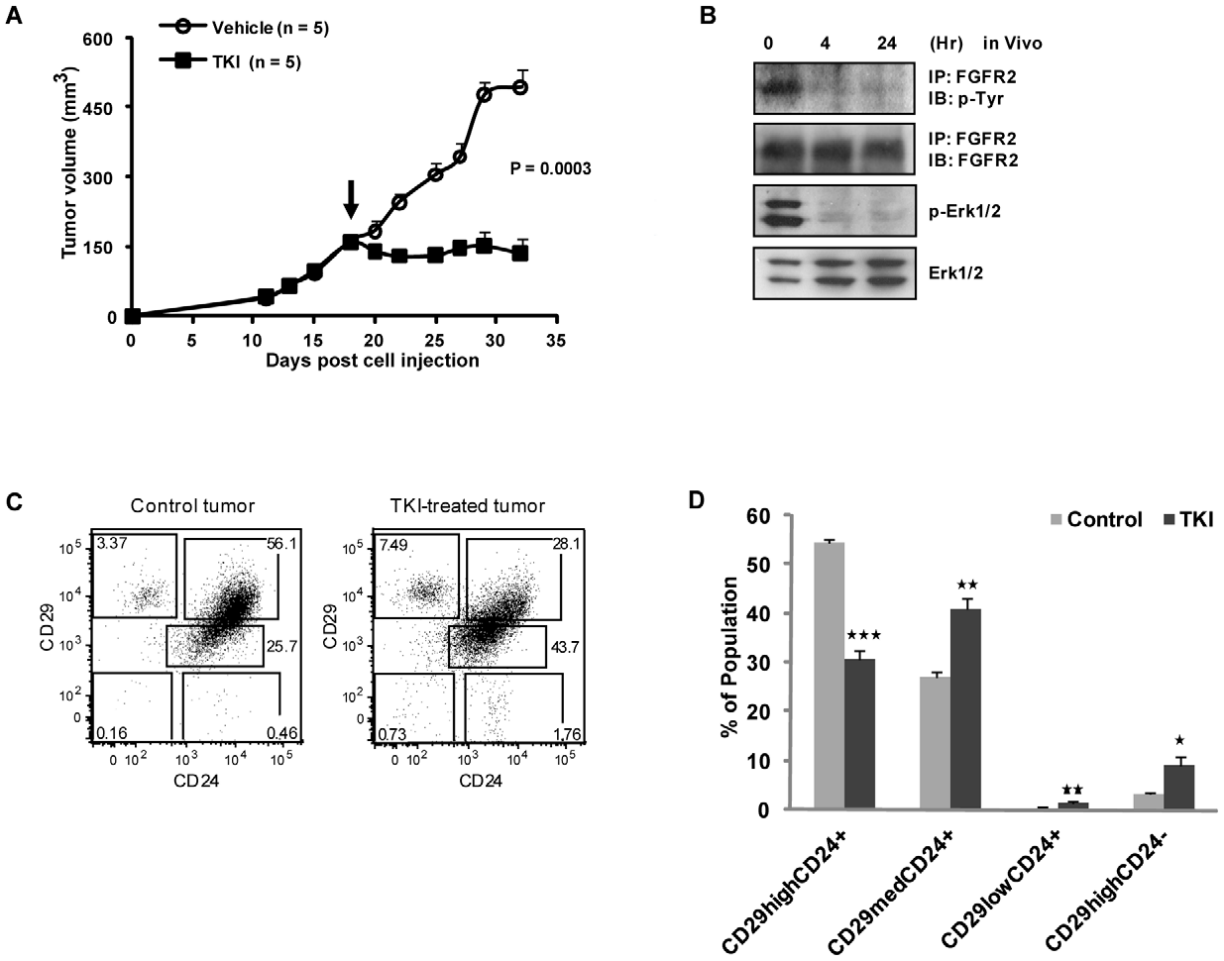
Antitumor Activity of FGFR Inhibitor as a Result of a Decrease in the Breast TIC Subpopulation In Vivo. (A) Antitumor activity of FGFR inhibitor, TKI258. Daily oral administration of TKI258 at 50 mg/kg was initiated in tumor-bearing NOD/SCID mice (n = 5 per group) when the MMTV-PyMT breast tumors reached ∼150 mm^3^ in volume. *P* value at day 32 is indicated. (B) Reduced phosphorylation of FGFR2 and Erk1/2 upon treatment of breast tumors with TKI258. Breast tumors were collected at predose, 4 and 24 hours after administration of a single oral dose of TKI258. Tumors were homogenized, immunoprecipitated for FGFR2 and immunoblotted with anti-phosphotyrosine to evaluate phosphorylation of FGFR2. The membranes were reprobed to evaluate total level of FGFR2 protein. (C) Flow cytometry analysis of CD24 and CD29 expression for TIC and non-TIC subpopulation frequencies in TKI258- or control-treated breast tumors. (D) Quantification of TIC and non-TIC subpopulations in TKI258- or control-treated breast tumors determined by flow cytometry analysis (C). Data (n = 3) represent mean ± SEM. Statistical comparison with control treatment (*P<0.04; **P<0.02; ***P<0.002).

To investigate whether inhibition of FGFR2 kinase activity would suppress the growth of breast tumors and TICs, we treated breast tumors with TKI258 (formerly CHIR258), an inhibitor of receptor tyrosine kinases including FGFRs [42]–[46]. Treatment of PyMT breast tumors with TKI258 resulted in growth suppression of tumors (Figure 6A). The growth inhibition by TKI258 *in vivo* was accompanied by significantly reduced phosphorylation of FGFR2 and Erk1/2 (Figure 6B), indicating the dependence of breast tumor growth on activation of FGFR2 and its downstream targets. To determine whether the inhibited growth of tumors treated with FGFR inhibitor was due to reduced numbers of TICs, we analyzed TIC and non-TIC subpopulations in TKI258-treated breast tumors using flow cytometry. Treatment of tumors with the FGFR inhibitor *in vivo* resulted in a ∼44% decrease in the TIC subpopulation (CD29^high^CD24^+^) compared to the control group (Figure 6C and 6D). In contrast, the treatment with the FGFR inhibitor led to an increase in the non-TIC subpopulations (∼34% increase in CD29^med^CD24^+^, ∼66% in CD29^low^CD24^+^, and ∼64% in CD29^high^CD24^−^ subpopulations) (Figure 6C and 6D). These results suggest that inhibition of FGFR2 kinase activity can suppress the growth of breast tumors probably by blocking the self-renewing capacity of TICs.

### Human Breast TICs Were Found Enriched in a FGFR2+ Population that Was Sufficient to Initiate Tumor Growth

**Figure 7:**
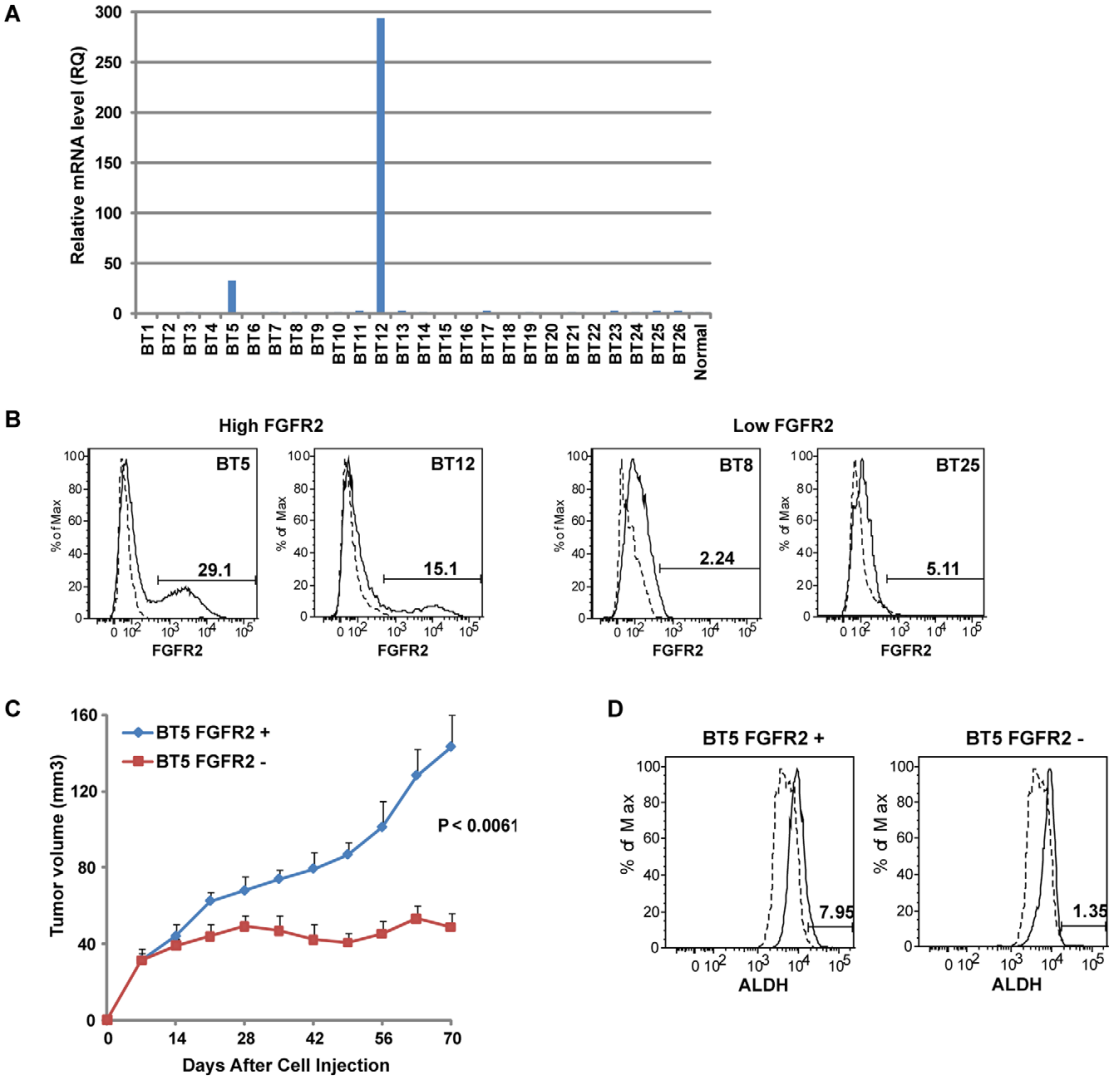
Human Breast TICs Were Enriched in FGFR2+ Population that Was Sufficient to Initiate Tumor Growth. (A) The expression levels of FGFR2 mRNA in patient-derived breast tumors. Quantitative real-time PCR was performed using cDNA generated from RNA isolated from 26 primary human breast cancer specimens. cDNA isolated from a normal breast tissue was used to normalize data and generate RQ. (B) Flow cytometry analysis of FGFR2 protein expression in primary human breast tumors. High (BT5 and BT12) or low level (BT8 and BT25) of FGFR2 protein corresponded to high or low level of *FGFR2* mRNA (A). This figure represents a typical result of three independent experiments. Dashed line shows an unstained control for each tumor sample. (C) Effect of FGFR2 expression on primary human breast tumor growth. FGFR2−overexpressing primary human breast tumor cells (BT5) were FACS sorted based on the expression of FGFR2. The two isolated populations (FGFR+ and FGFR2−) were injected in the mammary fat pad of the NOD/SCID mice (n = 5 per group). *P* value at day 70 is indicated. Data represent mean ± SEM. (D) Flow cytometry analysis of ALDH activity in FGFR2+ and FGFR2− subpopulations of BT5 tumors. Higher ALDH activity was found in the FGFR2+ population compared to the FGFR2− population. Dashed line shows a specific inhibitor of ALDH (DEAB)-treated labeling.

Thus far, we demonstrated the role of FGFR2 in the maintenance of mouse breast TICs. We next explored whether FGFR2 might play a role in maintaining a stem-like TIC pool in human breast cancer. First, we examined expression levels of FGFR2 in patient-derived breast tumors. Quantitative real-time PCR revealed a strong increase (32- to 293-fold) in *FGFR2* mRNA levels in 2 of 26 breast tumors (∼8%) compared to a normal breast sample (Figure 7A). Furthermore, flow cytometry analysis confirmed that high (BT5 and BT12) or low level (BT8 and BT25) of FGFR2 protein (Figure 7B) corresponded to high or low level of *FGFR2* mRNA (Figure 7A).

To investigate whether primary human breast tumor cells with FGFR2 overexpression are functionally dependent on FGFR2, FGFR2-overexpressing primary human breast tumor cells (BT5) were FACS-sorted based on the expression of FGFR2. These two isolated populations (FGFR2+ and FGFR2−) of human breast tumor cells were injected in the mammary fat pad of NOD/SCID mice. The population of tumor cells with no FGFR2 expression (FGFR2−) displayed significantly slower tumor growth as compared to the FGFR2-expressing tumor cells (FGFR2+) (Figure 7C). We also investigated whether the FGFR2+ population is enriched for human breast tumor-initiating cells by monitoring aldehyde dehydrogenase (ALDH) activity since elevated ALDH activity has been shown in normal and malignant human mammary stem cells [47], [48]. Flow cytometry analysis revealed ∼6-fold higher ALDH activity in the FGFR2+ population compared to the FGFR2- population, indicating that human breast TICs were enriched in the FGFR2+ population that was sufficient to initiate tumor growth (Figure 7D). This result suggests that FGFR2 may play important roles in the proliferation and survival of human breast tumor-initiating cells.

## Discussion

Many different types of tumors contain a subset of stem-like tumor-initiating cells, which drive tumor initiation and recurrence. Thus, therapeutic strategies that selectively target TICs are proposed to lead to the development of successful anti-cancer therapies. The molecular mechanisms underlying tumor initiation and stem-like function of TICs, however, are poorly understood. To understand these mechanisms, we investigated to identify essential regulators for the maintenance of breast TICs. We first identified and characterized TICs and non-TICs isolated from a mouse breast cancer model. We demonstrated that the breast TICs, which are highly enriched in the CD29^high^CD24^+^ subpopulation, drive tumor initiation and possess stem-like capabilities including self-renewal, heterogeneous differentiation, and bipotency, compared with other subpopulations. Comparison of gene expression between breast TICs and non-TICs revealed that FGFR2 is upregulated in the breast TICs. Our studies suggested that FGFR2 is essential in sustaining the breast TIC pool through promotion of self-renewal and maintenance of bipotency of TICs. Our studies contribute to the characterization of stem-like function of breast tumor-initiating cells and to the understanding of the associated molecular mechanisms. In addition, our results targeting FGFR2 suggest that there are specific targets that may be used to successfully eliminate TICs in human cancers.

This is the first report demonstrating convergence of two recent findings: FGFR2 function in breast cancer and in normal mammary stem cell maintenance. *FGFR2* was recently identified as a risk factor in breast cancer from several genome-wide association studies for breast cancer [32], [39]–[41]. Indeed, *FGFR2* is amplified and overexpressed in 4–12% of human breast cancers [49]–[51]. A mouse mammary tumor virus (MMTV) insertional mutagenesis screen for genes associated with mammary cancer also identified *FGFR2* and *FGF10* [52]. Independent of these roles of FGFR2 in breast cancer, recent studies in mammary gland development revealed that FGFR2 is essential in the maintenance of terminal end buds (TEBs) where mammary stem cells are active during mammary gland development [37], [38]. Although independent studies demonstrated that FGFR2 plays a significant role in normal or malignant breast tissue, none of these findings made FGFR2 signaling as a functional link between the normal mammary stem cells and malignant mammary stem cells.

It has been speculated that the self-renewal regulators in normal stem cells may be shared by malignant stem cells or TICs, since malignant stem cells have been proposed to derive from the transformation of normal stem or progenitor cells through dysregulated self-renewal [4]. The potential role of FGFR2 in mammary stem cell maintenance in TEBs [37], [38] is further supported by the requirement of FGF10–FGFR2IIIb signaling for embryonic and postnatal mammary gland development [53], [54]. In addition, a role for FGFR2 in stem cell maintenance is reinforced by the evidence of FGFR2 functions in a variety of stem cells. These functions include self-renewal and proliferation of the undifferentiated state of multipotent trophoblast stem cells during embryogenesis [55]–[57], proliferation of osteogenic stem cells [58], and maintenance of self-renewal and undifferentiated growth of human embryonic stem cells [59].

Activating somatic mutations of *FGFR2* have been reported in some cancers including lung squamous cell carcinoma, gastric cancer, cervical carcinoma, and endometrial carcinoma, implicating its role in cancer development [60]–[62]. We sequenced the regions of exons (exon 3, 6, 7, 8, 9, 12, and 14) in which somatic mutations of *FGFR2* have been reported in the other carcinoma cases [60], [63]. Although we found one SNP, rs1047100 (696A→G) in exon 6 of *FGFR2* in 60% of the 30 primary human breast tumors analyzed, we failed to detect any of the previously described *FGFR2* somatic mutations in either 30 primary human breast tumors or in MMTV-PyMT tumors (unpublished data), suggesting that *FGFR2* somatic mutations may be an infrequent event in breast cancer. However, several recent genome-wide association studies demonstrated that germ-line polymorphisms in intron 2 of *FGFR2* are associated with breast cancer susceptibility [32], [39]–[41] emphasizing the importance of *FGFR2* in breast cancer. The mechanism by which *FGFR2* functions as a risk factor in breast cancer, however, remains unknown. The present findings that FGFR2 is an essential regulator for the maintenance of breast TICs may answer questions, at least in part, in breast cancer.

Alternative splicing in the third immunoglobulin-like domain of FGFR2 mRNA results in the formation of FGFR2IIIb and FGFR2IIIc isoforms (Figure S4A). This differential splicing determines the ligand specificity of FGFR2 in a tissue specific manner [64], [65]. Epithelial cells express FGFRIIIb, whereas mesenchymal cells express FGFR2IIIc (Figure S4A). To investigate which isoform of FGFR2 is present in MMTV-PyMT tumors, we sequenced the cDNAs generated from mRNA regions common to both FGFR2IIIb and FGFR2IIIc isoforms (Figure S4A and S4B). This analysis revealed that MMTV-PyMT tumors express FGFR2IIIb isoform which is exclusively expressed by epithelial cells. Further studies will be required to understand a functional link between the FGF ligands (to the particular FGFR2 isoform) and the FGFR2 roles in TIC maintenance.

TKI258 is an orally bioavailable kinase inhibitor of the FGFRs. Its activity against VEGFR and PDGFR, along with the FGFR inhibitory activity, is responsible for the potent anti-angiogenic component of the compound. TKI258 is now being investigated in phase III and II clinical trials in renal cell carcinoma, breast cancer, myeloma and urothelial cancer. In our studies we have shown that use of TKI298 leads to an inhibition of tumor growth in this mouse model of breast cancer and this data could serve as a predictor for its use in breast cancer patients. Given the multitargeted nature of TKI258, our results emphasize the need to develop FGFR2 specific inhibitors.

Our gene expression comparison between breast TICs and differentiated non-TICs led to the identification of upregulated genes in breast TICs, including *FGFR2*, *FOXA1*, *GABRA4*, *SDC4*, *CSF3*, *ETNK1*, and *CDC42BPG*. These genes have been shown to be involved in mammary carcinogenesis, development and stem cell activities [30], [31], [33]–[36]. However, the expression of these genes has not been previously investigated regarding their involvement in maintenance of tumor-initiating cells. Further studies will also be required to understand any functional link between FGFR2 and other genes that were upregulated in TICs. It is possible that the inhibition of other TIC-specific genes along with FGFR2 inhibition produces synergistic anti-tumor activity, more effective at eradicating tumor initiating cells.

## Materials and Methods

### Mouse Strain, Clinical Samples, and Tumor Cell Preparation

MMTV-PyMT mice (FVB/n strain) [19], NOD.CB17-Prkdc^scid^ and NOD.Cg-Prkdc^scid^ Il2rg^tm1WJ1^/SzJ mice were bred and maintained according to institutional guidelines [the Genomics Institute of the Novartis Research Foundation (GNF) Institutional Animal Care and Use Committee]. The GNF Biomedical Institutional Review Board approved this specific study, and the breast cancer specimens were handled and maintained according to protocols approved by GNF Biomedical Institutional Review Board, ensuring that samples were delivered to GNF with unique identifiers and GNF had no access to any personal patient information, other than pre-existing pathology provided by Asterand (Detroit, MI). Establishment of primary human tumor models for in vivo validation studies was performed in accordance with the animal protocols (P06-147 and P09-243) approved by the GNF Institutional Animal Care and Use Committee for this specific study. Primary tumors were isolated from 3- to 6-month-old MMTV-PyMT mice, and were mechanically dissociated and digested in serum-free mammary epithelial growth media (MEGM, Lonza, Walkersville) containing 200–300 U/ml collagenase (Worthington). The resulting tumor suspension was treated with 0.25% trypsin for 1–2 min and 1× RBC (red blood cell) lysis buffer for 3 min, and filtered to obtain single tumor cells.

### Cell Staining and Flow Cytometry

Hematopoietic and endothelial cell lineages from the tumor cells were depleted by staining with antibodies against CD45 and/or TER119 and CD31. In most cases, FACS-sorted CD45^−^ tumor cells contained less than ∼0.4% of hematopoietic (TER119^+^) and endothelial (CD31^+^) cell lineages. Otherwise, tumor cells were stained with antibodies against TER119-biotin (BD Pharmingen) and CD31-biotin (BD Pharmingen) to deplete hematopoietic and endothelial cell lineages by magnetic-activated cell sorting (MACS). Tumor cells were labeled with antibodies against antigens including CD29-APC (BioLegend), CD24-FITC (BD Pharmingen), CD45-PE-Cy7 (BD Pharmingen), CD45-biotin, FGFR2–PE (R&D Systems), FGFR2 (Sigma), FGFR2–PE (R&D Systems), and were stained with ALDEFLUOR for ALDH activity (StemCell Technologies) according to the manufacturer's instructions. For xenografted human tumors, cells were labeled with anti-mouse H-2k^d^–biotin (BD Pharmingen) antibody to deplete mouse cells by MACS magnetic separation columns (Milteni Biotec). Cells were resuspended in 0.5 µg/ml propidium iodide (PI) to label dead cells and sorted on FACSDiva or FACSAria (Becton Dickinson). Other flow cytometry analyses were done in BD LSR II flow cytometer (Becton Dickinson).

### Drug Administration

TKI258 solution was formulated in water freshly every day. Daily oral administration of TKI258 at 50 mg/kg was initiated in tumor-bearing NOD/SCID mice when mammary tumors reached ∼150 mm^3^ in volume. The tumors were measured every 2–4 days.

### Transplantation of Breast Tumor Cells

For limiting dilution transplantation of tumor cells, sorted primary or secondary MMTV-PYMT tumor cells were counted in decreasing numbers (5000 to 5 cells) and resuspended in MEGM media with 50% matrigel, and injected into the mammary fat pads of 4- to 6-week-old NOD.CB17-Prkdc^scid^ or NOD.Cg-Prkdc^scid^ Il2rg^tm1WJ1^/SzJ mice. Ten thousand transduced MMTV-PYMT tumor cells with lentiviral shRNAs and retroviral cDNAs were injected. Eighty thousand tumor cells of two sorted populations (FGFR+ and FGFR2−) from primary human breast tumor BT5 were injected into the mammary fat pad of the NOD/SCID mice. The tumors were measured once a week. Secondary and tertiary tumors were harvested when the size of those tumors reached the size similar to primary tumors that were isolated from 3- to 6-month-old MMTV-PyMT mice.

### Cell Culture, Mammosphere assay and Viral Transduction

Sorted MMTV-PyMT tumor cells were cultured in modified MEGM media supplemented with 20 ng/ml bFGF (Invitrogen), 20 ng/ml EGF (Invitrogen), 0.5× B27 (Invitrogen), 4 µg/ml heparin (Sigma), 2 mM glutamine (Invitrogen), 5 µg/ml insulin (Sigma), 10^−6^ M hydrocortisone (StemCell Technologies), 0.2% BSA (Sigma), and 1–2% FBS. Bovine pituitary extract was excluded from MEGM kit. Monolayer culture of primary tumor cells was performed in Primaria plates (Becton Dickinson). For Mammosphere (>50 µm in diameter) assays, two or four thousand sorted cells, or ten thousand transduced cells were cultured in ultra-low attachment plates (Corning) with serum-free MEGM media and counted 2–3 weeks after seeding. For differentiation conditions, the same number of FACS-sorted cells from various populations was cultured on collagen-coated plates (Becton Dickinson) in the presence of 5–10% FBS. Tumor cells were spin-transduced with concentrated virus and were selected by 0.8–1 µg/ml puromycin 24 hr after infection.

### Proliferation and Soft Agar Assay

Proliferation of tumor cells was determined by cell viability using CellTiter-Glo reagent (Promega). Three thousand transduced cells were plated in 96-well Primaria plates for cell viability assays. To determine the anchorage-independent growth, five thousand or ten thousand tumor cells were seeded in a top layer of 0.3% agar in MEGM with supplements that was placed on the top of a bottom layer of 0.6% agar in DMEM without any supplement in 12-well plate. Colonies (>100 µm in diameter) were counted 3 weeks after seeding.

### Immunofluorescence and Quantification

PTA-fixed cells and formalin-fixed paraffin-embedded tumor sections were stained with antibodies against Cytokeratin 18 (Abcam), Cytokeratin 14 (Covance), smooth muscle actin (SMA) (Sigma), and FGFR2 (Santa Cruz). While one representative image per group was presented, at least three images from the different positions in the plate per group were collected using the ImageXpress Ultra laser scanning confocal microscope (Molecular Devices). Image quantification was achieved using the multi-wavelength cell scoring analysis module, and an average of three values from three images (different positions) per group was calculated.

### Statistics and Limiting Dilution Analyses of Tumors

Limiting dilution analyses of tumors were performed using the limdil function of the ‘statmod’ package (http://bioinf.wehi.edu.au/software/elda/) for the R statistical programming environment (http://www.r-project.org). 95% confidence intervals were computed for tumor-initiating cell frequencies. The single hit Poisson assumption was validated by likelihood ratio tests and accepted for all dilution series (*P*>0.05) [66]. To evaluate the difference between groups, the Student's t test was used. Differences of *P*<0.05 were considered significantly different.

### RNA Isolation and Quantitative Real-Time PCR (qRT-PCR)

Total RNA from tumor cells was extracted with the RNeasy kits (QIAGEN) or TRIzol (Invitrogen), according to the manufacturer's instructions. cDNA was prepared using the SuperScript II or III reverse transcriptase (Invitrogen) and subjected to qRT-PCR on 7700HT Fast real-time PCR system (Applied Biosystems) using gene-specific primers. qRT-PCR was performed with either Taqman (Applied Biosystems) or SYBR Green (Applied Biosystems). Gene expression was normalized by b-actin (endogenous control) and calibrator sample using the ΔCt method to calculate RQ (relative quantity). The difference between the threshold cycle of a target and the threshold cycle of the endogenous control was calculated as ΔCt = Ct(target)−Ct(endogenous control). The difference between the average ΔCt value of a target sample and the average ΔCt for the calibrator sample was calculated as ΔΔCt(test sample) = AvgΔCt(test sample)−AvgΔCt(calibrator sample). Expression fold value (RQ, Relative Quantity) is calculated as RQ = 2^-ΔΔCt^. The following Taqman Gene Expression Assays (Applied Biosystems) were used: mouse *FGFR2* (Mm00438941_m1), human *FGFR2* (Hs01552926_m1), mouse *ACTB* (beta actin) endogenous control (4352341E), human *ACTB* (beta actin) endogenous control (4326315E). The following primer sets were used with SYBR Green: *FGFR2*, 5′-GAGAAGGAGATCACGGCTTC-3′ and 5′-AAGTCTGGCTTCTTGGTCGT-3′; *FOXA1*, 5′-ATGAGAGCAACGACTGGAACA-3′ and 5′-TCATGGAGTTCATAGAGCCCA-3′; *SDC4*, 5′-GTCCCCGGAGAGTCGATTC-3′ and 5′-GCACCAAGGGCTCAATCACTT-3′; *GABRA4*, 5′-ACAATGAGACTCACCATAAGTGC-3′ and 5′-GGCCTTTGGTCCAGGTGTAG-3′; *CSF3*, 5′-ATGGCTCAACTTTCTGCCCAG-3′ and 5′-CTGACAGTGACCAGGGGAAC-3′; *ETNK1*, 5′-CTGTTCACAGATGGGATCACAA-3′ and 5′-CGCGGAAACTTTTCACTTCCTC-3′; *CDC42BPG*, 5′-GGCCTTGGCTACTAAGATGGC-3′ and 5′-TTGGCACGGATCTCAGCTTC-3′.

### Analysis of FGFR2 Isoform

For analysis of FGFR2 isoform present in MMTV-PyMT tumors, the cDNA synthesized from RNA isolated from MMTV-PyMT primary tumors was amplified by PCR using the specific primer pairs: primer 448 and primer 1021; primer 530 and primer 1021. 448, 5′-AGCTGGACTGCCTGCAAATGCCTCCA-3′; 530, 5′-ATGTGGAGTTTGTCTGCAAGGTTTA-3′; 1021; 5′-AGCTGGACTCGGCCGAAACTGTTA-3′. The PCR product was sequenced for analysis of FGFR2 isoform.

### Microarray Analysis

RNeasy kits (QIAGEN) or TRIzol (Invitrogen) was used to extract total RNA from tumor cells, according to the manufacturer's instructions. The integrity and concentration of RNA were determined via microfluidic analysis on an Experion instrument (BioRad). GeneChip Two-Cycle Target Labeling Assays (Affymetrix, Santa Clara, CA) were used to amplify cRNA from 40 ng total RNA. Standard Affymetrix protocols were used to process Affymetrix Mouse430_2 microarrays (Affymetrix, Santa Clara, CA). All CEL file images were processed as a single group using gcRMA. The microarray data were serially sorted by fold changes of expression values in the TIC population compared to the other three non-TIC populations in MMTV-PyMT mammary tumors, The data sets were then filtered by three categories following; (1) as the maximum intensity of expression values among four populations is greater than 100, (2) as the fold change of expression values in the CD29^high^CD24^+^ population compared to CD29^low^CD24^−^ population is greater than 4, and (3) as the minimum intensity of expression values among two samples of CD29^high^CD24^+^ population is greater than 17. This analysis led to the identification of genes that are significantly upregulated in TICs compared to non-TICs. The microarray data described will be deposited in NCBIs Gene Expression Omnibus (http://www.ncbi.nlm.nih.gov/geo/) and will be accessible through a GEO Series accession number.

### Lentiviral shRNAs and Retroviral cDNA Constructs

Lentiviral shRNAs targeting FGFR2 (shFGFR2) were constructed within lentivirus plasmid vector pLKO.1-Puro (Sigma). Two different short hairpin RNAs targeting different regions of *FGFR2* were used. The target regions of shFGFR2s are following: shFGFR2-2, GCCAGGGATATCAACAACATA; shFGFR2-3, GCATCGCATTGGAGGCTATAA. We performed rescue experiments by ectopically expressing FGFR2 (constructed within Su-VGIP3 retrovirus plasmid vector) resistant to shRNA knockdown.

### Immunoblotting and Immunoprecipitation

Tumors were homogenized, and tumor cells were lysed on ice, immunoprecipitated for FGFR2 and immunoblotted with anti-phosphotyrosine to evaluate phosphorylation of FGFR2. The membranes were reprobed to evaluate total level of FGFR2 protein. The following antibodies were used for immunoblotting: FGFR2 (Sigma or Santa Cruz Biotech), p44/42 MAP Kinase (Erk1/2) (Cell Signaling Technology), phospho-p44/42 MAP Kinase (p-Erk1/2) (Cell Signaling Technology), β-actin (Cell Signaling Technology), phosphor-tyrosine (R&D Systems), and phospho-FRS2-α (Cell Signaling Technology).

## Supporting Information

Figure S1 Gating Strategy to Isolate Five Subpopulations and Purity of Sorted Populations in MMTV-PyMT Breast Tumors. Five populations were gated based on the compensation that is verified by single color staining of each maker and isolated by FACS. The bottom panel demonstrates that the purity of sorted populations was 80–100%.
(TIF)

Figure S2 Enrichment of Breast Tumor-Initiating Cells in CD29^hi^CD24^+^ Population. Comparison of the growth of secondary breast tumors that were driven from purified primary tumor cells of different subpopulations is shown. The number of cells injected is indicated next to the population. Limiting dilution analyses of tumors were performed in NOD/SCID mice.
(TIF)

Figure S3 Loss of FGFR2 Led to a Reduced Number of Bipotent Precursor-like TICs In Vivo. (A) Immunofluorescence of shNT−, shFGFR2−, shFGFR2 + FGFR2 (rescue construct)-transduced primary MMTV-PyMT breast tumors for K18 and K14. Paraffin-embedded tumor sections were stained for luminal epithelial marker (K18, green) and myoepithelial marker (K14, red), and DAPI (nuclei, blue). Magnifications of the boxed regions are shown in the two rows below each figure. The scale bars represent 34 µm. (B) Immunofluorescence of shNT−, shFGFR2−, shFGFR2+FGFR2-transduced primary MMTV-PyMT breast tumors for SMA. Tumor sections were stained for myoepithelial marker (SMA, red) and DAPI (nuclei, blue). The scale bars represent 34 µm. (C) Flow cytometry analysis of CD24 and CD29 expression for breast TIC and non-TIC subpopulation frequencies in shNT−, shFGFR2−, shFGFR2+FGFR2–transduced primary MMTV-PyMT breast tumors.
(TIF)

Figure S4 MMTV-PyMT Breast Tumors Express FGFR2IIIb Isoform. (A) Structure of FGFR2 isoforms, FGFR2IIIb and FGFR2IIIc, generated from alternative splicing of FGFR2 mRNA. Epithelial cells express FGFRIIIb utilizing exon 8, whereas mesenchymal cells express FGFR2IIIc including exon9. PCR primers were designed for the mRNA region spanning from 5′ exon 8 (primer 448 or primer 530) and to 3′ exon 9 (primer 1021) that is common to both FGFR2IIIb and FGFR2IIIc isoforms. Ig, immunoglobulin-like; TM, transmembrane domain; TK, tyrosine kinase domain. (B) Analysis of FGFR2 isoform present in MMTV-PyMT breast tumors. The cDNA synthesized from RNA isolated from MMTV-PyMT primary breast tumors was amplified by PCR using the specific primers (A). Primer pairs used for PCR include primer 448 and primer 1021 (left lane); primer 530 and primer 1021 (right lane). Only one PCR product was obtained from each PCR reaction and was sequenced.
(TIF)

Figure S5 CD29^high^CD24^+^ Cells Have Self-Renewal Capacity and Contain Bipotent Precursor-like Cells. Differentiation potential of breast tumor subpopulations assessed by immunofluorescence. A lower magnification (scale bars = 65 µm) from figure 2B is shown to include more cells in the bigger area. The breast tumor cells from the four FACS-sorted subpopulations were cultured under the differentiation condition. The sorted cells from various populations were plated at the same cell density on collagen-coated plates. The differentiated cells were stained for the luminal epithelial marker (K18, green), the myoepithelial markers (K14 and SMA, red), and DAPI (nuclei, blue). A significant portion of CD29^high^CD24^+^ cells contains K18^+^K14^+^ (bipotent precursor-like), whereas the majority of cells from the other subpopulations contain lineage-restricted cells.
(TIF)

Figure S6 Inhibition of Oncogenic Signaling by Loss of FGFR2. Inhibition of downstream target activation upon FGFR2 knockdown. Primary MMTV-PyMT breast tumor cells were transduced with lentiviral short hairpin RNAs (shRNAs) targeting FGFR2 (shFGFR2). The shFGFR2-4 efficiently knocked down the expression of FGFR2 protein and inhibited phosphorylation of Erk1/2 and FRS2, as evidenced by immunoblotting with anti-phospho-ERK1/2 (p-ERK1/2) and anti-phospho-FRS2 (p-FRS2). The shFGFR2-1 that partially knocked down the FGFR2 expression had little effect on ERK or FRS2 phosphorylation. The membranes were reprobed for actin and Erk1/2 as loading controls.
(TIF)
