# Supplementary figures and images for: FGFR2 Promotes Breast Tumorigenicity through Maintenance of Breast Tumor-Initiating Cells
Source: PLoS One. 2013 Jan 2;8(1):e51671. doi: 10.1371/journal.pone.0051671 (PMC3534701; doi:10.1371/journal.pone.0051671)

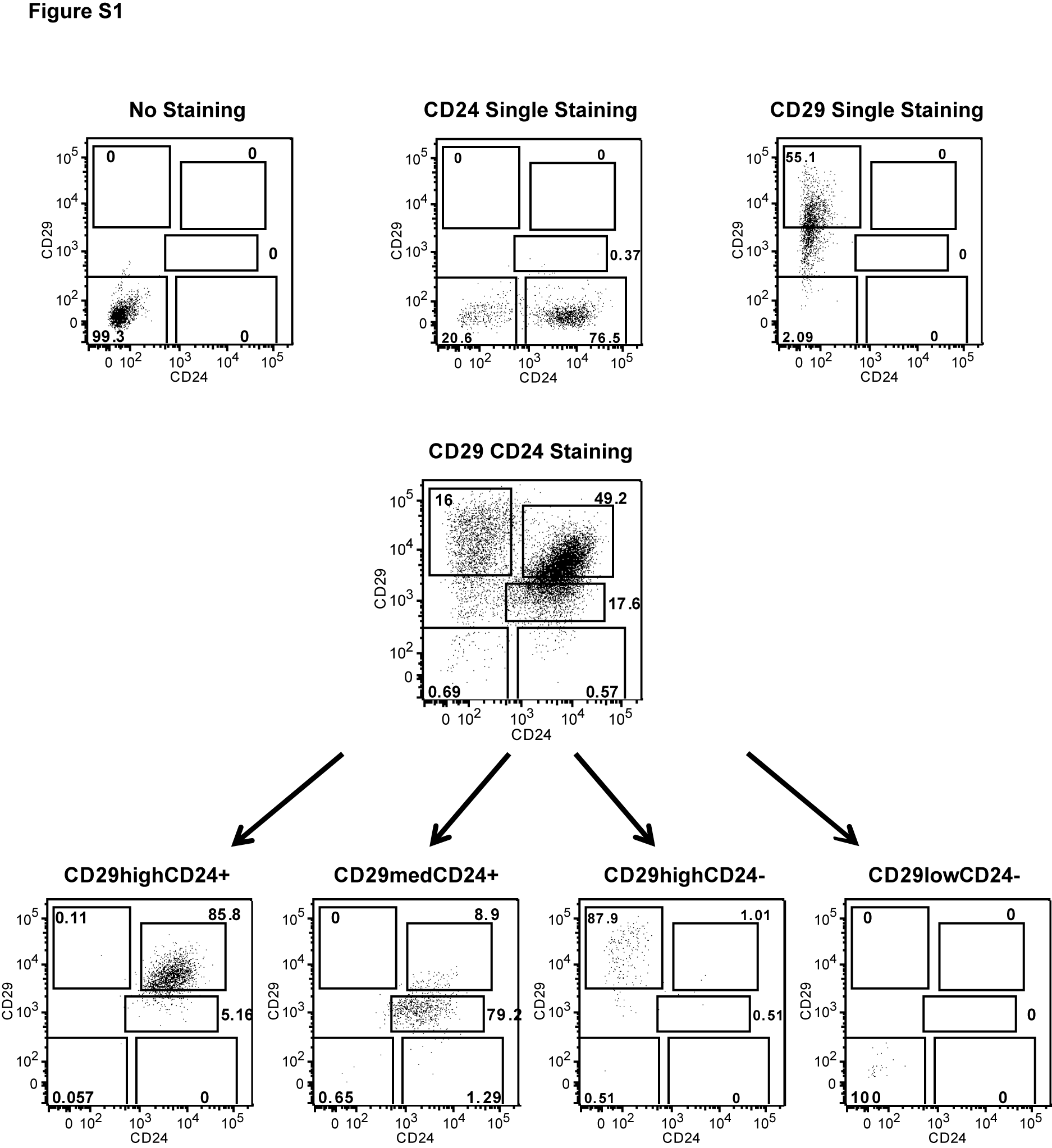

Supplement: Figure S1 — Gating Strategy to Isolate Five Subpopulations and Purity of Sorted Populations in MMTV-PyMT Breast Tumors. Five populations were gated based on the compensation that is verified by single color staining of each maker and isolated by FACS. The bottom panel demonstrates that the purity of sorted populations was 80–100%. (TIF) [file pone.0051671.s001.tif]

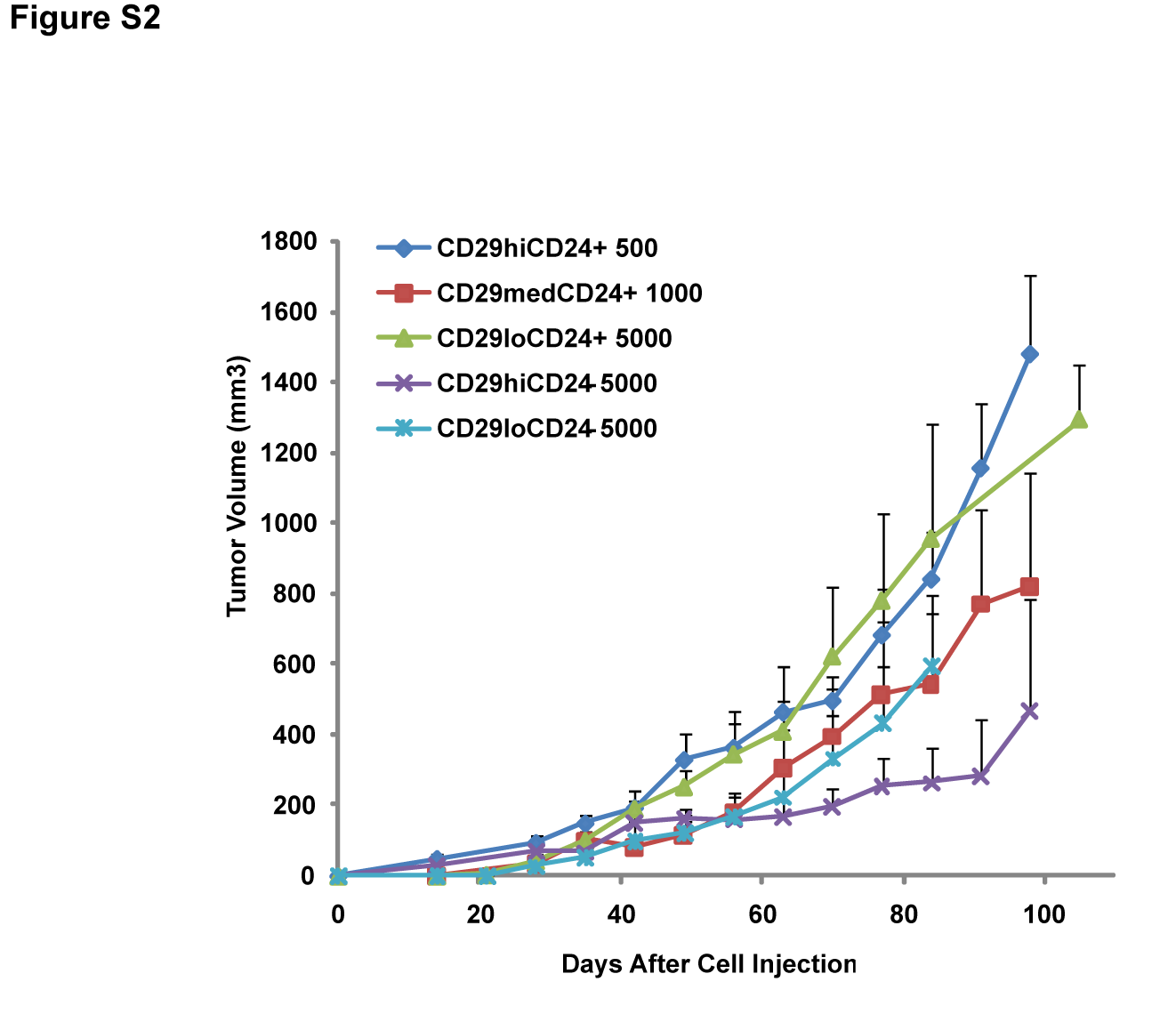

Supplement: Figure S2 — Enrichment of Breast Tumor-Initiating Cells in CD29hiCD24+ Population. Comparison of the growth of secondary breast tumors that were driven from purified primary tumor cells of different subpopulations is shown. The number of cells injected is indicated next to the population. Limiting dilution analyses of tumors were performed in NOD/SCID mice. (TIF) [file pone.0051671.s002.tif]

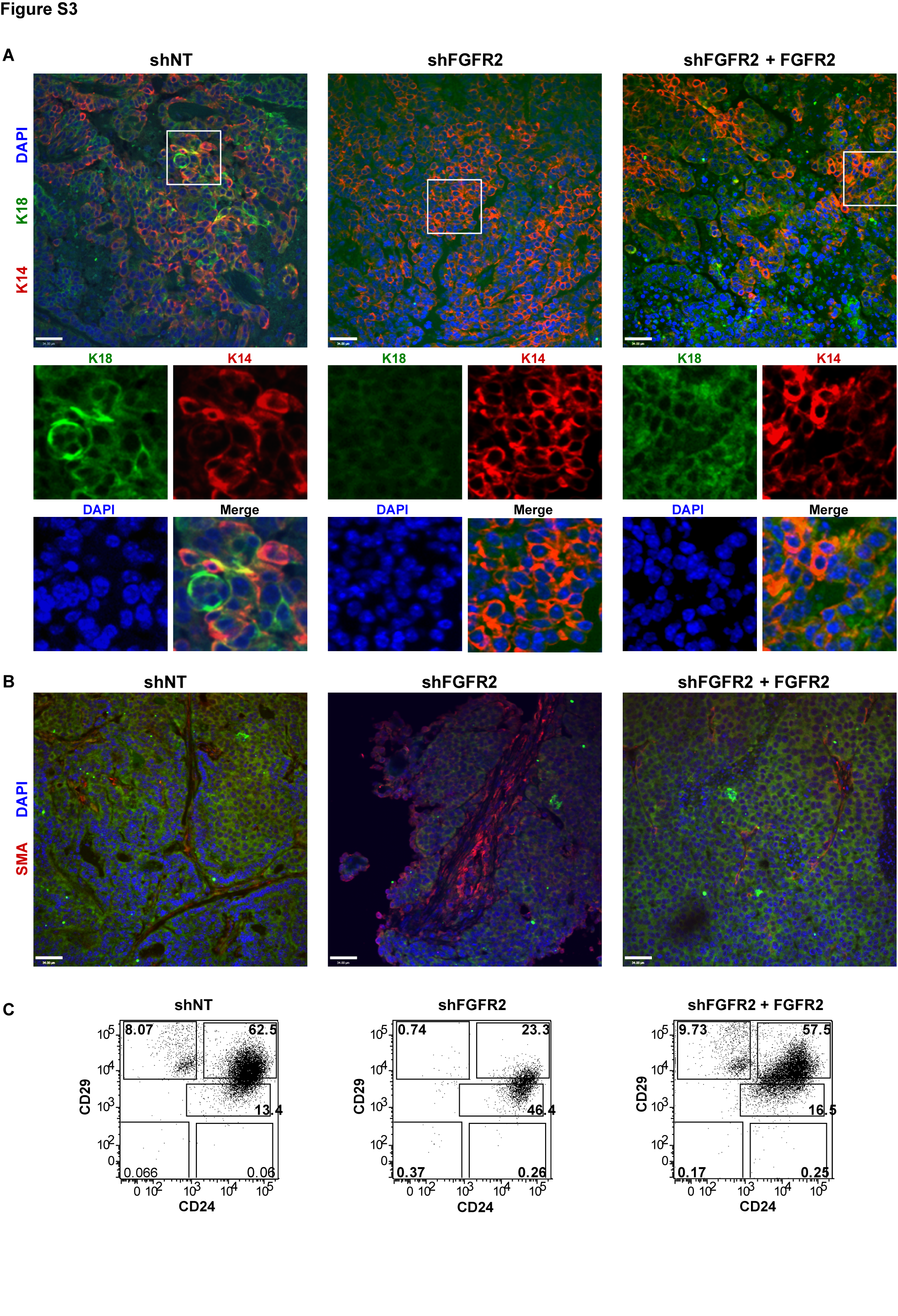

Supplement: Figure S3 — Loss of FGFR2 Led to a Reduced Number of Bipotent Precursor-like TICs In Vivo. (A) Immunofluorescence of shNT−, shFGFR2−, shFGFR2 + FGFR2 (rescue construct)-transduced primary MMTV-PyMT breast tumors for K18 and K14. Paraffin-embedded tumor sections were stained for luminal epithelial marker (K18, green) and myoepithelial marker (K14, red), and DAPI (nuclei, blue). Magnifications of the boxed regions are shown in the two rows below each figure. The scale bars represent 34 µm. (B) Immunofluorescence of shNT−, shFGFR2−, shFGFR2+FGFR2-transduced primary MMTV-PyMT breast tumors for SMA. Tumor sections were stained for myoepithelial marker (SMA, red) and DAPI (nuclei, blue). The scale bars represent 34 µm. (C) Flow cytometry analysis of CD24 and CD29 expression for breast TIC and non-TIC subpopulation frequencies in shNT−, shFGFR2−, shFGFR2+FGFR2–transduced primary MMTV-PyMT breast tumors. (TIF) [file pone.0051671.s003.tif]

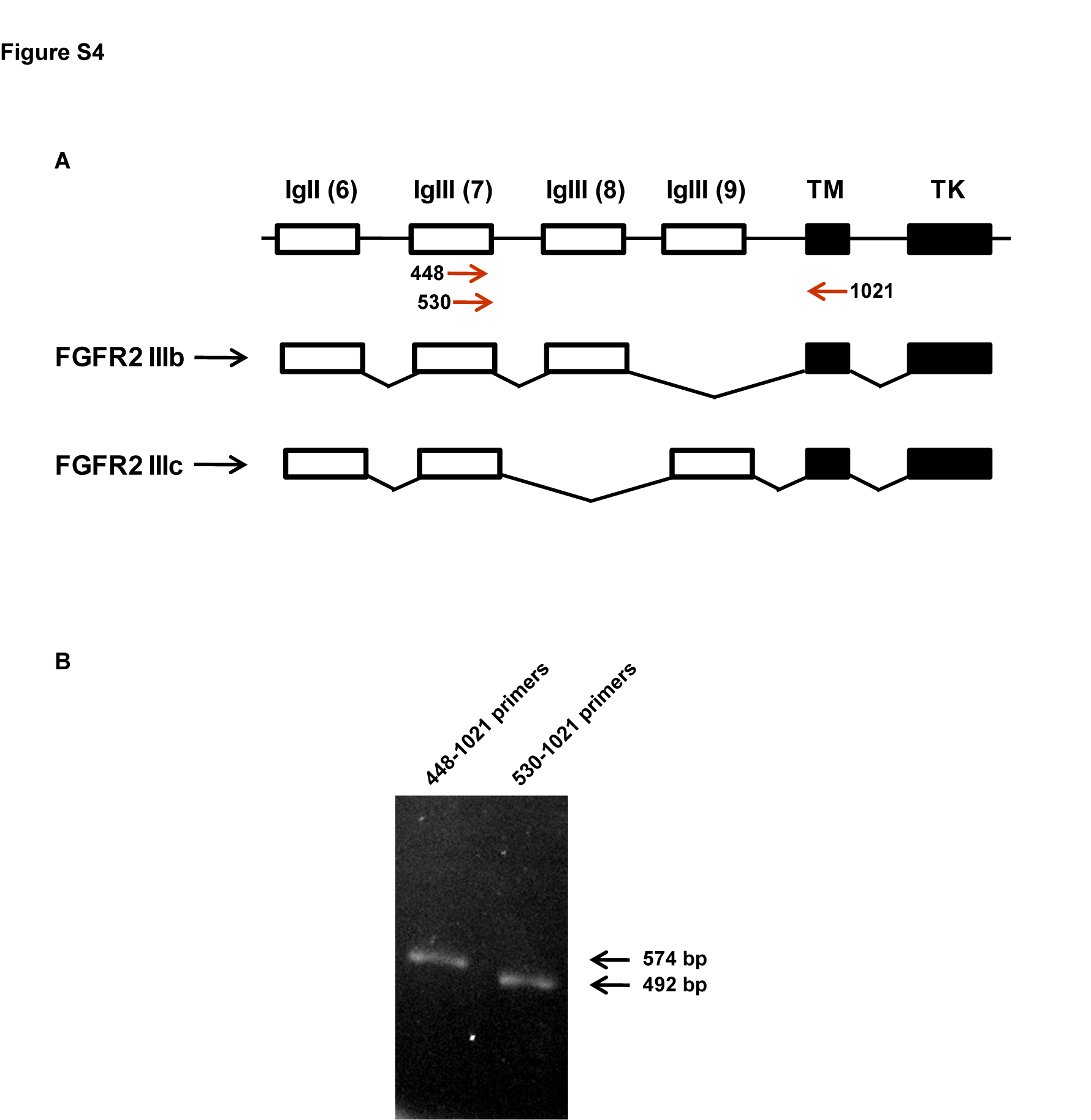

Supplement: Figure S4 — MMTV-PyMT Breast Tumors Express FGFR2IIIb Isoform. (A) Structure of FGFR2 isoforms, FGFR2IIIb and FGFR2IIIc, generated from alternative splicing of FGFR2 mRNA. Epithelial cells express FGFRIIIb utilizing exon 8, whereas mesenchymal cells express FGFR2IIIc including exon9. PCR primers were designed for the mRNA region spanning from 5′ exon 8 (primer 448 or primer 530) and to 3′ exon 9 (primer 1021) that is common to both FGFR2IIIb and FGFR2IIIc isoforms. Ig, immunoglobulin-like; TM, transmembrane domain; TK, tyrosine kinase domain. (B) Analysis of FGFR2 isoform present in MMTV-PyMT breast tumors. The cDNA synthesized from RNA isolated from MMTV-PyMT primary breast tumors was amplified by PCR using the specific primers (A). Primer pairs used for PCR include primer 448 and primer 1021 (left lane); primer 530 and primer 1021 (right lane). Only one PCR product was obtained from each PCR reaction and was sequenced. (TIF) [file pone.0051671.s004.tif]

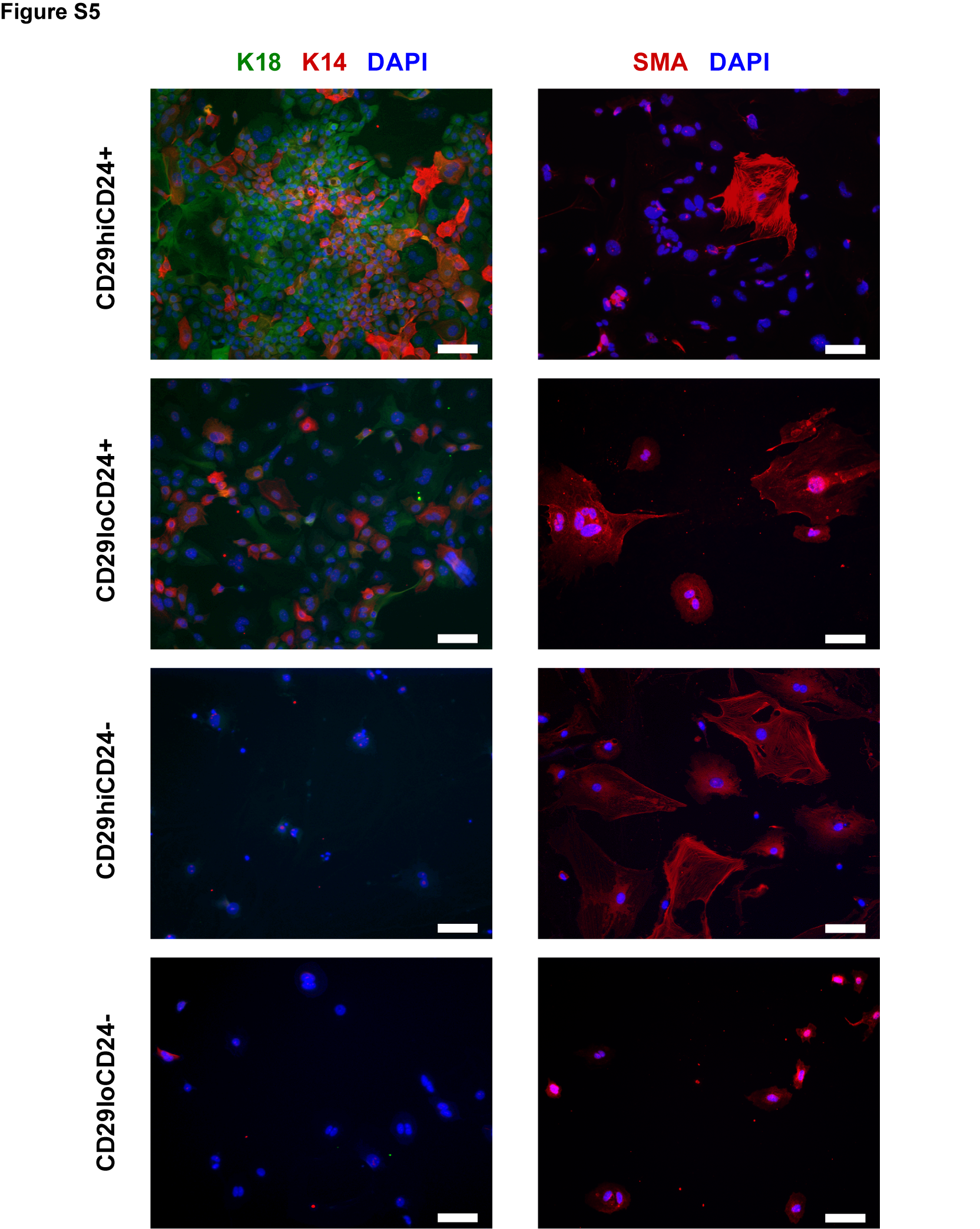

Supplement: Figure S5 — CD29highCD24+ Cells Have Self-Renewal Capacity and Contain Bipotent Precursor-like Cells. Differentiation potential of breast tumor subpopulations assessed by immunofluorescence. A lower magnification (scale bars = 65 µm) from figure 2B is shown to include more cells in the bigger area. The breast tumor cells from the four FACS-sorted subpopulations were cultured under the differentiation condition. The sorted cells from various populations were plated at the same cell density on collagen-coated plates. The differentiated cells were stained for the luminal epithelial marker (K18, green), the myoepithelial markers (K14 and SMA, red), and DAPI (nuclei, blue). A significant portion of CD29highCD24+ cells contains K18+K14+ (bipotent precursor-like), whereas the majority of cells from the other subpopulations contain lineage-restricted cells. (TIF) [file pone.0051671.s005.tif]

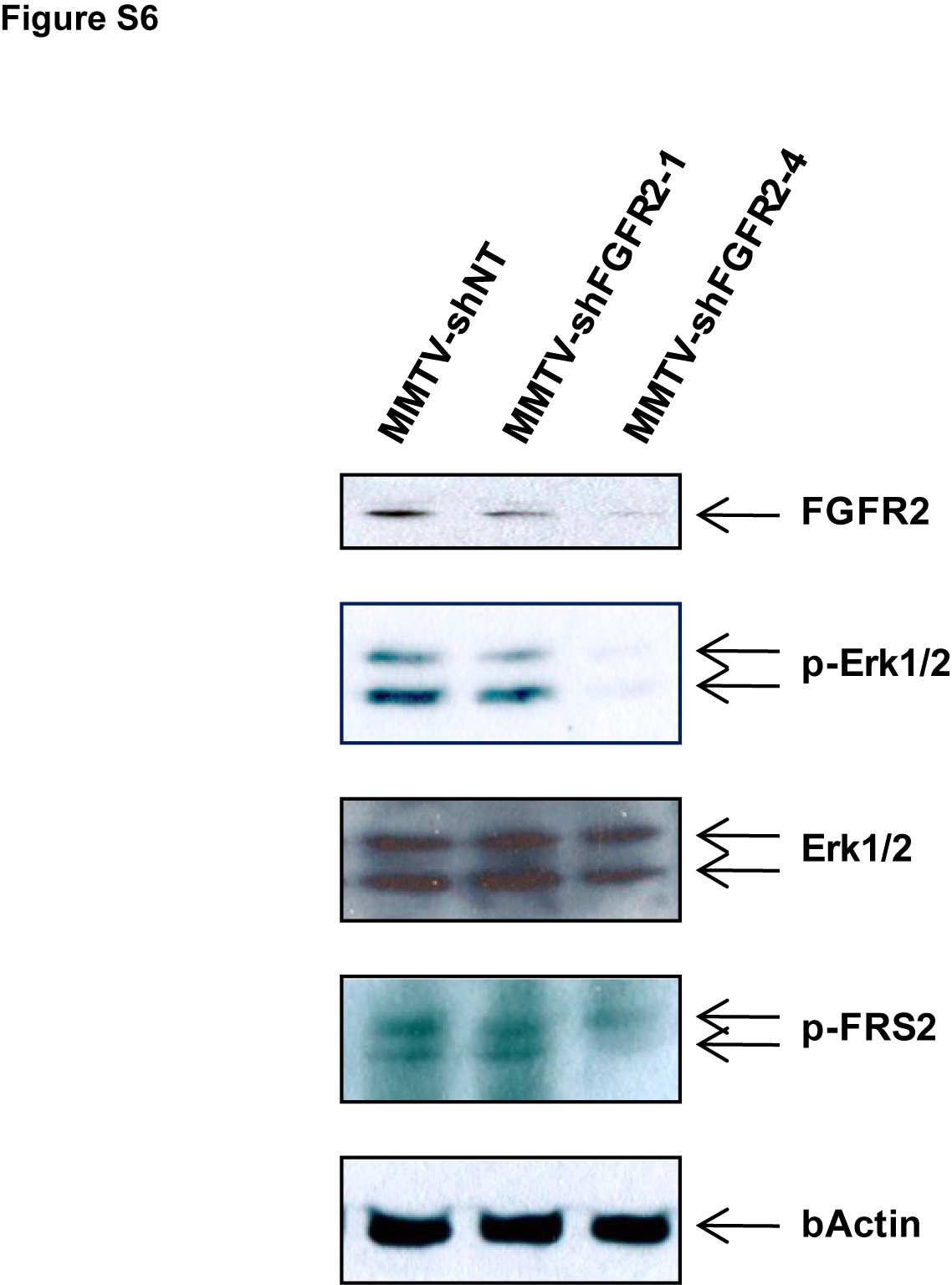

Supplement: Figure S6 — Inhibition of Oncogenic Signaling by Loss of FGFR2. Inhibition of downstream target activation upon FGFR2 knockdown. Primary MMTV-PyMT breast tumor cells were transduced with lentiviral short hairpin RNAs (shRNAs) targeting FGFR2 (shFGFR2). The shFGFR2-4 efficiently knocked down the expression of FGFR2 protein and inhibited phosphorylation of Erk1/2 and FRS2, as evidenced by immunoblotting with anti-phospho-ERK1/2 (p-ERK1/2) and anti-phospho-FRS2 (p-FRS2). The shFGFR2-1 that partially knocked down the FGFR2 expression had little effect on ERK or FRS2 phosphorylation. The membranes were reprobed for actin and Erk1/2 as loading controls. (TIF) [file pone.0051671.s006.tif]
